# Axonal injury following mild traumatic brain injury is exacerbated by repetitive insult and is linked to the delayed attenuation of NeuN expression without concomitant neuronal death in the mouse

**DOI:** 10.1111/bpa.13034

**Published:** 2021-11-03

**Authors:** Yasuaki Ogino, Tytus Bernas, John E. Greer, John T. Povlishock

**Affiliations:** ^1^ Department of Anatomy and Neurobiology Virginia Commonwealth University School of Medicine Richmond Virginia USA; ^2^ Department of Neurosurgery Virginia Commonwealth University School of Medicine Richmond Virginia USA; ^3^ Department of Surgery Hunter Holmes McGuire Veterans Affairs Medical Center Richmond Virginia USA

**Keywords:** axonal injury, mild traumatic brain injury, NeuN, neuronal death, p‐c‐Jun, repetitive traumatic brain injury

## Abstract

Mild traumatic brain injury (mTBI) affects brain structure and function and can lead to persistent abnormalities. Repetitive mTBI exacerbates the acute phase response to injury. Nonetheless, its long‐term implications remain poorly understood, particularly in the context of traumatic axonal injury (TAI), a player in TBI morbidity via axonal disconnection, synaptic loss and retrograde neuronal perturbation. In contrast to the examination of these processes in the acute phase of injury, the chronic‐phase burden of TAI and/or its implications for retrograde neuronal perturbation or death have received little consideration. To critically assess this issue, murine neocortical tissue was investigated at acute (24‐h postinjury, 24hpi) and chronic time points (28‐days postinjury, 28dpi) after singular or repetitive mTBI induced by central fluid percussion injury (cFPI). Neurons were immunofluorescently labeled for NeuroTrace and NeuN (all neurons), p‐c‐Jun (axotomized neurons) and DRAQ5 (cell nuclei), imaged in 3D and quantified in automated manner. Single mTBI produced axotomy in 10% of neurons at 24hpi and the percentage increased after repetitive injury. The fraction of p‐c‐Jun+ neurons decreased at 28dpi but without neuronal loss (NeuroTrace), suggesting their reorganization and/or repair following TAI. In contrast, NeuN+ neurons decreased with repetitive injury at 24hpi while the corresponding fraction of NeuroTrace+ neurons decreased over 28dpi. Attenuated NeuN expression was linked exclusively to non‐axotomized neurons at 24hpi which extended to the axotomized at 28dpi, revealing a delayed response of the axotomized neurons. Collectively, we demonstrate an increased burden of TAI after repetitive mTBI, which is most striking in the acute phase response to the injury. Our finding of widespread axotomy in large fields of intact neurons contradicts the notion that repetitive mTBI elicits progressive neuronal death, rather, emphasizing the importance of axotomy‐mediated change.

## Graphical abstract

The pathogenesis of mild traumatic brain injury, which has been linked to morbidity with high social costs, remains little known, with some advocating neuronal death versus diffuse axotomy. Using sophisticated and comprehensive imaging approaches within large neocortical regions, we demonstrated that neither singular nor repetitive injuries evoked neuronal death, despite parallel alterations of NeuN expression, and increased levels of axotomy in both the acute and chronic phases. These findings indicate that axotomy is the key pathological response to mild injury.

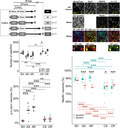

## 1 INTRODUCTION

Traumatic brain injury (TBI) is a major health care problem worldwide. In recent estimates, the global incidence of TBI is as high as 900 cases per 100,000 individuals [1] and today, millions are living with TBI‐related long‐term disabilities [2, 3, 4]. Mild TBI (mTBI) is the least severe type of TBI. It is associated with subtle or no alteration of consciousness and the absence of overt intracranial abnormalities, constituting 70%–90% of overall TBI cases [1, 5]. Despite the term “mild”, mTBI has been suggested to be linked to a variety of persistent sequelae potentially having significant impact on the daily life of the injured [6]. Recently, it has been recognized that certain sub‐populations who are frequently exposed to external impact to the head, via contact sports, military engagement, and domestic violence, are at higher risk for mTBI‐related symptoms. Accordingly, these observations have garnered increased public attention for mTBI and its ensuing dysfunction [7, 8]. To date, extensive research has shown that mTBI exerts subtle structural changes with its associated morbidity. However, the overall complexity of mTBI is yet to be revealed. Further complicating our appreciation of the pathobiology of mTBI is the recognition that repetitive mTBIs, a common event in patients, generally portends exacerbated structural and functional responses in comparison to a singular insult. Accordingly, recent research has accelerated on those phenomena associated with repetitive mTBI.

Our lab as well as others have examined repetitive mTBI in animals and have confirmed that repetitive injuries of specific intensity and temporal spacing exacerbate the pathological changes associated with a singular injury alone. These studies conducted in the acute phases of injury assessed axonal injury and its associated anterograde and retrograde sequelae as well as the potential for neuronal death [9, 10, 11, 12, 13, 14]. Unfortunately, the majority of the work published in this area has focused on the acute phase response to repetitive mTBI rather than its chronic assessment. Accordingly, the long‐term structural and functional changes associated with repetitive mTBI are poorly appreciated, particularly in the context of axotomy and its retrograde neuronal consequences [15, 16, 17]. Specifically, it remains unclear whether the axotomized neurons persist postinjury, undergo either hypertrophic or atrophic change or rather do they or other related neuronal populations die over a more chronic time course. Not only are these issues theoretically complex, but also, they present many technological challenges in attempting to achieve their resolution. The reliance on structural or molecular markers of injury presents many barriers due to the diffuse nature of mTBI. Further, the assessment of histological, histochemical and immunocytochemical neuronal markers of injury, together with the rigorous quantitative assessments, also pose significant challenges.

In the current investigation, we examine the acute and chronic consequences of singular and repetitive mTBI using a comprehensive and rigorous approach. To this end, we incorporate spinning disk confocal microscopy to rapidly examine multiple fluorophores in multiple samples from consistent neocortical regions, allowing neuronal numbers, signal intensity and volumetric measures to be critically assessed via 3D‐based image analysis algorithms. Following repetitive mTBI, we employ a widely used marker specific to axotomized neuronal soma, phosphorylated c‐Jun (Ser 63) (p‐c‐Jun), to quantitatively assess the axotomized neuronal population. This approach was interfaced with the use of NeuN to assess the overall burden of neuronal loss, with the parallel use of NeuroTrace, a fluorescent Nissl stain dye to provide potential confirmatory information on neuronal survival in the neocortical areas evaluated. This approach was predicated on the recent observation that antibodies to NeuN may not represent the full number of existing neurons [12]. Using large‐volume data sets obtained from the same neocortical regions of differently injured animals, we confirmed that repetitive mTBI resulted in an increased burden of axotomy as evidenced by increased number of p‐c‐Jun profiles. Despite these rather dramatic axotomy‐mediated responses, the related neocortical domains showed no evidence of neuronal cell death. Rather, TBI induced a divergent response with respect to NeuN expression within the axotomized and non‐axotomized neuronal populations. Collectively, these studies of repetitive mTBI do not support the potential for any injury‐related neuronal cell loss. Rather, they demonstrate that increased axotomy‐mediated changes are the most likely contributor to the resulting pathology and any associated morbidity.

## 2 MATERIALS AND METHODS

### 2.1 Animals

Adult male mice C57BL/6J (#000664, Jackson Laboratory, Bar Harbor, ME) (8–11 weeks) were obtained directly from the vendor and kept in house for 48 h to habituate prior to experimentation. Animals were housed in vivarium under a 12‐h light/12‐h dark cycle with free access to food and water. The ambient temperature of vivarium and surgery room were set at 22.2℃ and maintained within the range consistent with the *Guide for the Care and Use of Laboratory Animals: 8th Edition*. All experimental procedures were conducted during the light cycle. A total of 32 animals were subjected for experimentation after group assignment described below.

### 2.2 Experimental design

**FIGURE 1:**
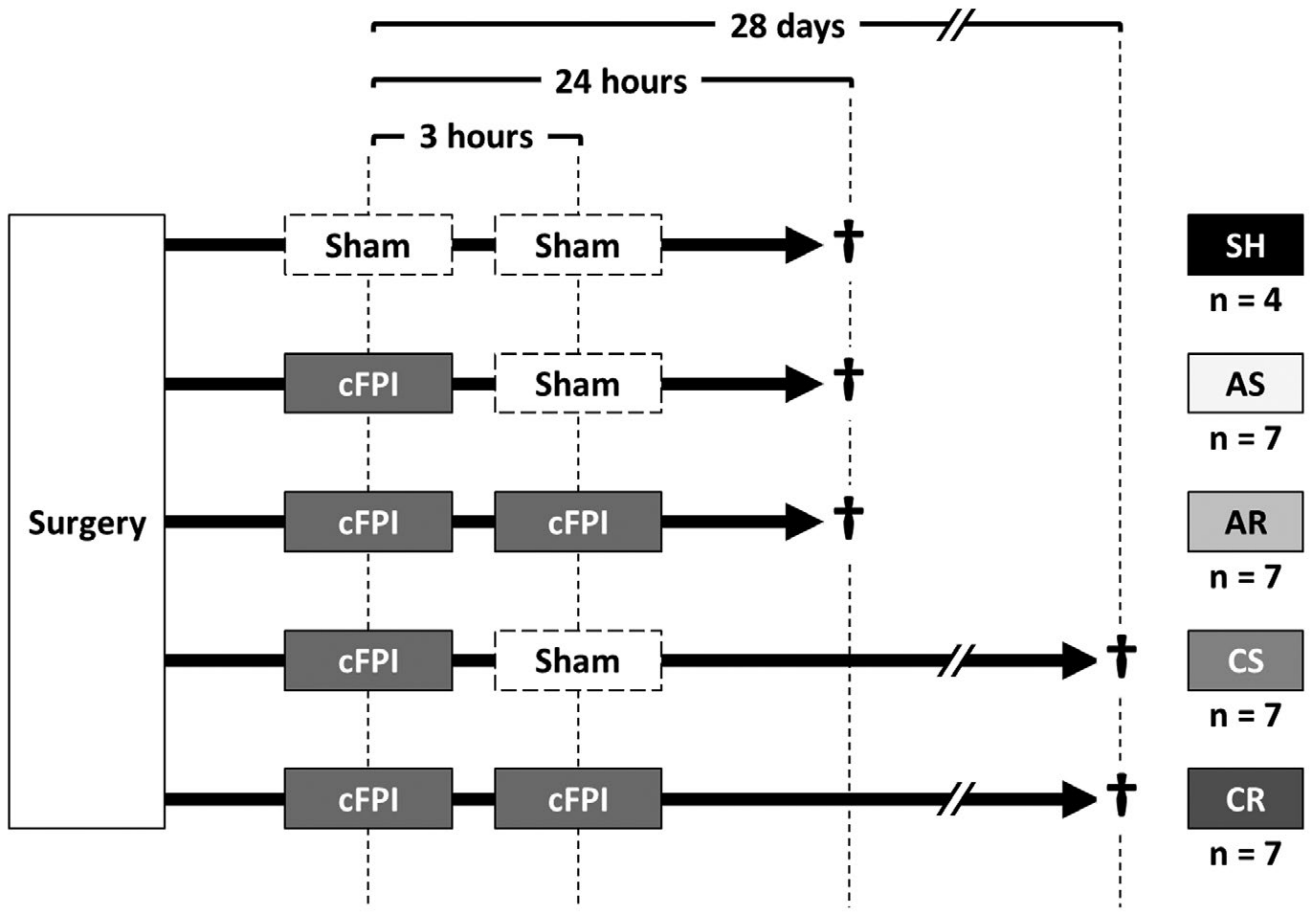
Animals were assigned to 5 experimental groups defined by the number of injuries (none, single or repetitive) and survival time after the first injury (24hpi or 28dpi). Two experimental TBIs were given at a 3‐h interval in a form of cFPI at 1.6 atm, an intensity consistent with mild TBI

The overall experimental design is summarized in Figure 1. Animals were randomly assigned into one of the following 5 experimental groups defined by the number of injuries and survival time after the first mTBI. These groups included a “Sham” group (“SH” group), an acute‐phase with single mTBI group (“AS” group), an acute‐phase with repetitive mTBI group (“AR” group), a chronic‐phase with single mTBI group (“CS” group) and a chronic‐phase with repetitive mTBI group (“CR” group). Experimental TBI was induced by central fluid percussion injury (cFPI). Three combinations of cFPI and/or sham injury were administered to the animals at an interval of 3 h. These included repetitive sham injuries (SH group), single cFPI followed by sham injury (AS and CS groups) or repetitive cFPI (AR and CR groups). From the initial mTBI, overall survival times of 24 h postinjury (24hpi) were employed for those in the SH, AS and AR groups while a 28‐day postinjury survival time (28dpi) was employed for those in the CS and CR groups, respectively. Sample size of each group was determined to achieve adequate statistical power to observe significant differences in the number of axotomized neurons at 24hpi between single and repetitive mTBI (i.e., between AS and AR) based on our previous experience [12]. A smaller sample size was assigned for the SH group because minimum histological change and minimum inter‐animal variability of data were anticipated. Accordingly, 4 animals were assigned to the SH group and 7 animals were assigned to each of the other experimental groups.

### 2.3 Surgical preparation and physiological assessments during surgery

Animals were subjected to surgical preparation for cFPI as described previously [12]. Briefly, 3 mm diameter craniectomy was prepared along the sagittal suture midway between bregma and lambda, then a 20‐gauge luer‐lock hub was placed and stabilized on the skull over the craniectomy site using cyanoacrylate and dental acrylic. All surgical procedures were performed sterilely under anesthesia, which was initially induced via inspiration of 4% isoflurane in 100% oxygen in an anesthesia chamber for 4 min and then maintained with flow of 2% isoflurane in 100% oxygen via a nose cone with the mouse fitted in a stereotaxic frame (David Kopf Instruments, Tujunga, CA). An adequate plane of anesthesia was ensured by checking the absence of toe pinch response.

Body temperature was maintained at 37ºC using thermostatically controlled heating pad with a rectal temperature sensor (Harvard Apparatus, Holliston, MA). Heart rate, respiratory ratio, and arterial blood oxygenation (SpO_2_) were monitored using thigh‐clump pulse oximeter sensor (MouseOx, STARR Life Sciences, Oakmont, PA). After surgery, animals were placed in a cage passively warmed with heating gel until their full recovery from anesthesia.

### 2.4 Experimental mild TBI and postinjury behavioral assessments

The animals were fully anesthetized with inspiration of 4% isoflurane in 100% oxygen in an anesthesia chamber for 4 min. Next, a luer‐lock hub installed on the animal's skull was connected with FPI device (Custom Design and Fabrication, Virginia Commonwealth University, Richmond, VA) using a connector tube filled with sterile saline. Details of the device are described elsewhere [18]. The pendulum position of the device was calibrated to target 1.6 atm, an injury intensity previously determined to be in the mild‐range [12, 19, 20, 21]. The actual intensity of the pressure wave impacting the animal's dural surface was measured by a transducer at the end of the device and displayed on an oscilloscope (Tektronix TDS 210, Tektronix Inc., Beaverton, OR). For sham injury, identical procedures were used except for the pendulum release. The time duration from taking fully anesthetized animal out from the chamber to the finish of the injury procedure was within seconds.

After cFPI, the animals were closely monitored until recovery of the righting reflex, and then returned to a cage. The loss of righting reflex (LORR) durations, an established measure for assessing the loss of consciousness in animals [22], were recorded at each time after the 1st injury (1st LORR) and the 2nd injury (2nd LORR). After the second cFPI, the skin around the injury site was sutured and animals were returned to the vivarium. Since the LORR required evaluation immediately following the traumatic event, induction of cFPI and LORR assessments were performed by the same examiner with appropriate blinding wherever possible.

### 2.5 Brain tissue preparation

At the designated time point of euthanasia, the animals were intraperitoneally injected with an overdose of pentobarbital and then transcardially perfused with heparinized saline followed by 4% paraformaldehyde in Millonig's buffer pH 7.4. The occipital and parietal bone were promptly removed, then brain was removed from the skull and immersed in the same fixative solution overnight, followed by replacement with Millonig's buffer in 4ºC. Coronally dissected brains between the level of the optic chiasm and the midbrain were embedded in agarose and then were cut using a vibratome (Leica VT1000S, Leica Biosystems, Nussloch, Germany) to obtain 40‐µm‐thick, serial floating slices directly below the craniectomy site (from −0.58 to −2.5 mm posterior to bregma), a region well known in this mild cFPI model to generate traumatic axonal injury (TAI) in the neocortical gray within the primary somatosensory cortex (S1) [12, 19, 20, 21]. Sections were collected in 24‐well plate and stored in Millonig's buffer pH 7.4 at 4ºC. Of total 48 sections, the caudal 24 sections were used for the analyses, dividing them into 6 sets of sections containing 4 sections spaced 240 µm apart. One randomly selected set was used in the following immunohistochemical staining.

### 2.6 Immunohistochemistry

An antibody to p‐c‐Jun (Ser 63), a marker of axotomized neuronal soma, was used to visualize axotomized neurons. To preclude the potential that any observed axonal damage led to cell death or a death independent of axotomy, we employed NeuN together with NeuroTrace, a florescent Nissl dye. For counterstaining nuclei, DRAQ5 was chosen and used for cell segmentation in the automated image processing employed in this study.

All samples were stained in the same one session employing randomization and blinding to preclude animal identity. Free‐floating sections placed in culture plates were washed with PBS then subjected to heat induced epitope retrieval (HIER) by being immersed in 10 mM sodium citrate buffer pH 8.5 with 0.05% Tween‐20 and warmed from outside of the plates for 10 min with heated water at a temperature of 80ºC [12, 20, 21]. After gradual cooling at room temperature, sections were incubated in blocking buffer (10% normal goat serum and 0.5% Triton X‐100 in PBS) for 1 h and then incubated with primary antibodies in a dilution buffer (1% normal goat serum and 0.5% Triton X‐100 in PBS) overnight at 4ºC temperature. The sections were then washed with a dilution buffer and incubated in secondary antibody solution (secondary antibodies in dilution buffer) for 2 h. After several washing with dilution buffer and PBS, sections were immersed in the counterstain solution for 2 h in room temperature. Sections washed with 0.1 M Na‐phosphate buffer were placed on plain glass slides individually and sealed with mounting medium.

Primary antibodies to the following antigens were utilized in the indicated dilutions; p‐c‐Jun (Ser63) (anti‐rabbit monoclonal IgG, clone SY0297, NBP2‐67471, 1:200, Novus, Littleton, CO), NeuN (anti‐mouse monoclonal IgG1 clone A60, MAB377, 1:500, Millipore, Billerica, MA). Fluorophore‐conjugated secondary antibodies were utilized in the indicated dilutions; goat‐derived anti‐rabbit‐IgG Alexa A568 (1:500, Thermo Fisher Scientific, Waltham, MA), goat‐derived anti‐mouse‐IgG1 Alexa A488 (1:500, Thermo Fisher Scientific). The mixture of the following dyes in PBS were utilized as counterstain solution with the indicated dilutions; NeuroTrace 435/455, a fluorescent Nissl stain (N‐21479, 1:100, Thermo Fisher Scientific), DRAQ5 (1:500, Thermo Fisher Scientific). VECTASHIELD Vibrance (Vector Laboratories, Burlingame, CA) was used as mounting medium.

### 2.7 Spinning disk confocal microscopy

To address the technically demanding need to rapidly acquire multiple fluorescently labeled images from a large number of samples covering a large neocortical region, image acquisition was performed using a spinning disk confocal microscope, an inverted fluorescence microscope (Axio Observer Z1, Carl‐Zeiss Microscopy GmbH, Jena, Germany) equipped with a spinning disk unit (CSU‐X1A, Yokogawa, Tokyo, Japan) and an electron multiplication charge coupled device (EMCCD) camera (Photometrics Evolve 512). Prior to the image acquisition, unified setting for gain and exposure time were determined for each channel (wavelengths of 405, 488, 561 and 639 nm) using randomly selected samples from experimental slide set. Image acquisition was then performed in random order and in blinded fashion as follows; the field of view was centered initially over the S1 along the dorsolateral edge of the hippocampus using a dry 10x objective lens (EC Plan‐Neofluar 10x/0.30), followed by adjustments to maintain centering midway between upper and lower limit of neocortical layer V with a dry 20x objective lens (Plan‐Apochromat 20x/0.8). Next two‐by‐two tiled, z‐stack images were captured, with voxel size of 0.67 × 0.67 × 0.44 µm. The tile sets were automatically stitched with ZEN 2.0 blue (Carl‐Zeiss Microscopy GmbH, Jena, Germany) to obtain images of 666.00 × 665.33 µm in size in the X‐Y plane. After acquisition, the region of interest (ROI) equaling 630 × 200 µm was chosen with its long axis paralleling the cortical laminae in layer V and VI. These were determined individually by visualizing the NeuN‐channel image. This ROI included pyramidal layer V which was chosen based on our previous experience with this model [12, 20]. On 4 sections from each animal, images of ROI on both hemispheres were captured, yielding total 8 tiled, composite images from each animal.

### 2.8 Cell detection and quantitative image analysis

The stitched image data were deconvolved (15–20 iterations) using nominal objective point spread function (PSF) and signal to noise ratio (SNR) of 25. The maximum likelihood (ML) deconvolution was implemented in Huyghens (version 18.04, Scientific Volume Imaging, Hilversum, Netherlands). The deconvolved 3D images were further processed and quantified using a custom‐built algorithm implemented in MATLAB (version R2018a, MathWorks, Natick, MA). First, background was estimated with grayscale opening (19‐pixel radius) for each color channel and subtracted from the deconvolved images. Threshold values of signal intensity for each marker were empirically determined by two expert observers using randomly selected images from the experimental image set. Voxels where the signal intensities were equal or greater than these static thresholds were considered positive and included in the respective channel masks. Voxel intensities below the threshold were set to zero. The first step of cell segmentation was conducted using the DRAQ5 channel where nuclei were isolated as sets of 27 voxels (8‐connected). Adjacent nuclei were split with the Euclidean distance (ED) transform of the initial masks, followed by watershedding. Cell masks were generated by thresholding NeuN and NeuroTrace channels, followed by constrained dilation of nuclear masks (up to 10 iterations) used as seeds. If no cellular mask corresponding to a nucleus was present owing to insufficient signal quality, the nuclear mask was slightly dilated by 5 voxels and used as a surrogate. The nuclear masks were then subtracted from their cell counterparts to form cytoplasmic masks. The cells which did not have the corresponding nuclei detected were segmented using NeuroTrace data (thresholding followed by watershedding).

Signal intensities within the respective masks were used to quantify p‐c‐Jun and NeuN present in whole cell body, nucleus and cytoplasm. First, cumulative and mean signal intensities of p‐c‐Jun were calculated. The former represents the total amount of p‐c‐Jun whereas the latter its concentration in the corresponding compartment. Next, cumulative signal intensity of NeuN was calculated for ROI to represent total amount of NeuN presenting within the region. Cumulative and mean intensity of NeuN were calculated for single neurons as well. Finally, subpopulations of neurons positive and negative for either p‐c‐Jun or NeuN were identified in distributions of the respective signal intensities and verified using randomly selected images.

Volumetric assessment was performed to evaluate any potential morphological change in related to cellular response to the burden of TAI. Volume of the masks, cellular components and p‐c‐Jun+ profiles were calculated from the respective counts multiplied by the voxel volume (0.196 µm^3^).

Lastly, relationships between the cellular volume and the occurrence of axotomy or the attenuation of NeuN expression were evaluated. Neurons were divided into the quintiles based on cellular volume within neuronal populations of each individual animal as follows; Ex‐S, extra‐small (the minimum‐20 percentile of cellular volume); S, small (20–40 percentile); M, middle (40–60 percentile); L, large (60–80 percentile); Ex‐L, extra‐large (80‐the maximum). The fractions of p‐c‐Jun+ neurons or NeuN+ neurons within whole neuronal population were compared between subgroups within each experimental group.

### 2.9 Cell screening strategy

Nissl staining is highly sensitive for neurons, however, it is also known to stain glial cells [23, 24], although they lack cytoplasmic reaction for the Nissl dye [25, 26, 27, 28, 29]. To minimize potential glial contamination, we employed cell screening on identified cells via two criteria based on volumetric data of individual cells. Those cells in which the cellular volume was below 231.77 µm^3^, equaling the 99‐percentile value of NeuroTrace−/NeuN− cells, or those cells for which the ratio of nuclear volume to cellular volume (nuclear‐cellular volume ratio, NCVR) exceeded 0.5 were considered glia and eliminated from the further analyses.

### 2.10 Statistical analysis

Unless specifically stated, data pooled from each animal were considered a statistical unit. Normality of the data distribution was analyzed by Shapiro‐Wilk test. Homogeneity of the data distribution was analyzed by the Brown–Forsthye test. Differences between two experimental groups were analyzed by Welch's test for parametric data or by Brunner‐Munzel test for nonparametric data. Pairwise comparisons between two different subgroups within the same group were analyzed by pairwise Welch's test for parametric data or by Wilcoxon's signed rank test for nonparametric data. Comparisons between pairs of proportional data were analyzed by Pearson's chi‐square test. Multigroup comparison of physiological background between groups was assessed by Kruskal‐Wallis test. For other multi‐group comparisons, statistical analyses were performed for comparisons between the SH group and each injured group, between single and repetitive injury groups of the same time points (i.e., AS vs AR and CS vs CR), and between acute‐injured and chronic‐injured groups of corresponding injury mode (i.e., AS vs CS and AR vs CR). Pairwise comparisons between two populations within each experimental group were also performed as needed. In these multi‐group comparisons, pooled p values were adjusted using Holm's method. All analyses were performed as two‐sided. P values below 0.05 were considered as significant. In addition, correlation coefficient *r* for numerical data and/or odds ratio (OD) for proportional data were calculated for each pair of comparison to evaluate effect size of different experimental settings on the differences in the results. Data was summarized as mean ± standard error of the means (SEM). All statistical analyses were performed using R software (version 3.6.2) [30] with the following add on packages; “brunnermunzel” [31], “compute.es” [32], “exactRankTests” [33], “ggplot2” [34], “Rmisc” [35], “vcd” [36, 37]. Full list of the used packages are shown in Table S1.

## 3 RESULTS

### 3.1 Physiological data, injury intensity and loss of righting reflex durations

Physiological data, injury intensity and duration of LORR are summarized in Table S2.

There were no significant differences in age and body weight at the time of the surgery. Animals were maintained in physiological homeostasis, with the maintenance of normal heart rate, respiratory rate, and oxygen saturation levels.

The intensity of the first cFPI for the AS, AR, CS and CR groups was 1.59 ± 0.00 atm, followed by 352 ± 17 s of LORR. The SH group, employed a sham injury, demonstrated 17 ± 2 s of LORR at this time point. With the second cFPI, the injury intensity for the AR and CR groups reached 1.58 ± 0.01 atm, followed by 290 ± 9 s of LORR. Consistent with our experimental design (Figure 1), the other 3 groups were subjected to sham injury at this time point and demonstrated 19 ± 2 s of LORR.

Overall, background parameters and physiological data during surgery were equal over all groups and the intensity of cFPI and duration of LORR were all consistent with our previous studies using the same mTBI model [12, 20, 21].

### 3.2 General histological observations

Gross histological findings of the AS and AR groups demonstrated features consistent with acute phase observations described in previous studies using a cFPI model of mild injury [12, 19, 20, 21]. Overall tissue integrity was well preserved. The dorsal neocortex underneath the craniectomy site did not demonstrate contusion or cavitation. The brain parenchyma was free from any overt hemorrhage or hematoma. Isolated petechial hemorrhage was occasionally observed in the subcortical layer and/or corpus callosum. As expected, the SH group was unremarkable. Overall histology of chronic‐injured groups appeared similar to the acute‐injured groups, with the exception that isolated petechial hemorrhage could no longer be visualized. In summary, only minimal histological changes were noted throughout all samples, consistent with the nature of mTBI.

**FIGURE 2:**
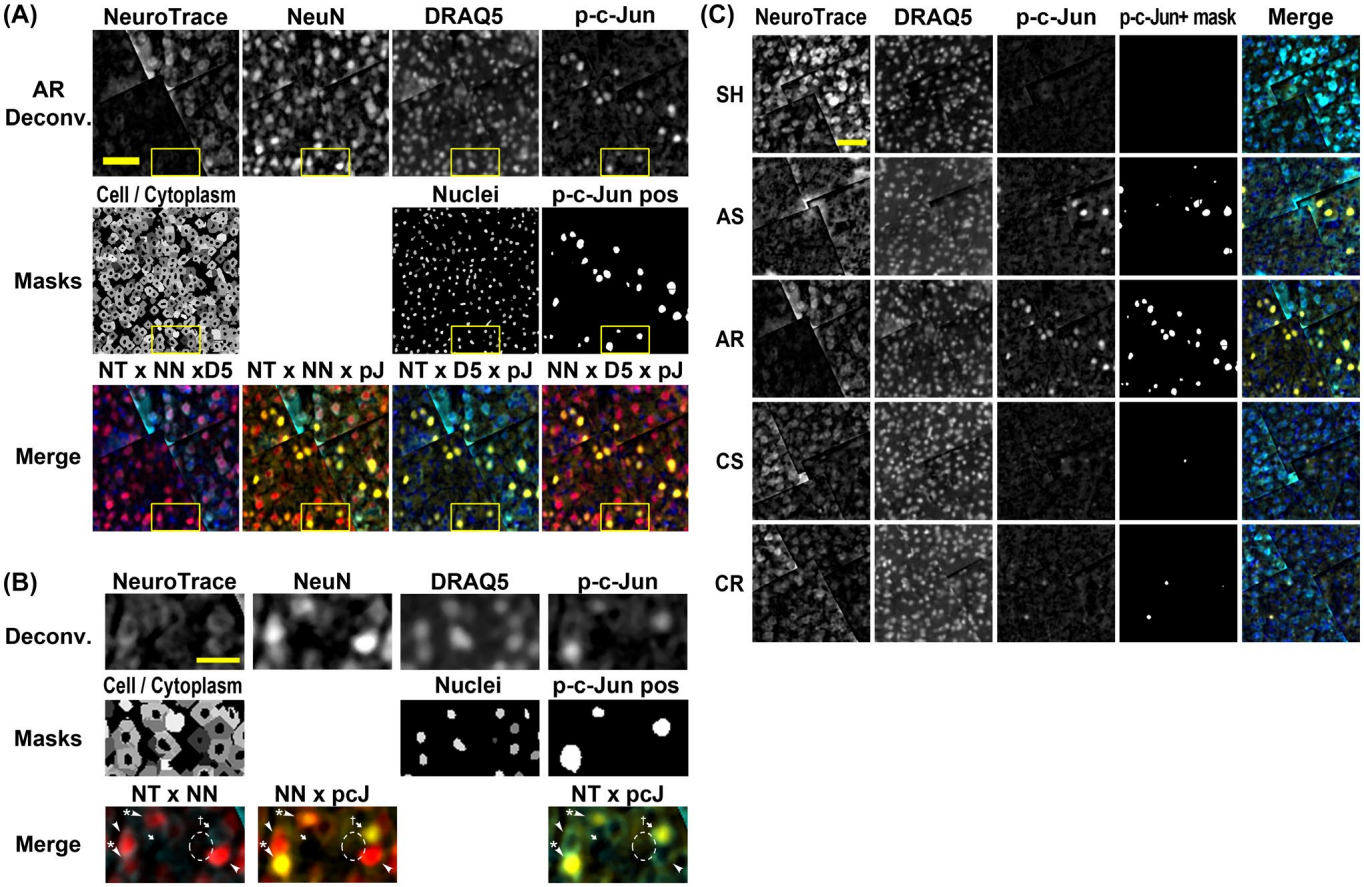
Representative images of immunofluorescently labeled cortical layer V. Tissues were labeled for NeuN and p‐c‐Jun and counterstained for neurons and nuclei with NeuroTrace (fluorescent Nissl dye) and DRAQ5. (A) Representative images from AR group after deconvolution (upper row), corresponding merged images (middle row) with the following color allocation; NeuroTrace (NT; cyan), NeuN (NN; red), p‐c‐Jun (pcJ; yellow) and DRAQ5 (D5; blue). Deconvolved images were further processed to generate cellular/cytoplasmic, nuclear and p‐c‐Jun+ masks (lower row). (B) Magnified images of the region indicated with yellow frame, showing cells differently labeled for the markers. NT+ NN+ cells including both p‐c‐Jun+ (arrowhead with asterisk) and p‐c‐Jun− cells (arrowhead) are most likely neurons. NT+ NN− cells are including both p‐c‐Jun+ (arrow with dagger) and p‐c‐Jun− (arrow). A cell negative for both NT and NN is also observed (dashed oval). Note that NT+ NN− cells are not necessarily neurons because NeuroTrace is known to stain glial cells, and thus the cellular screening was conducted before the analyses. (C) Representative images of each experimental group. AS and AR groups most frequently demonstrated p‐c‐Jun+ cells whereas Sham controls demonstrated few. CS and CR groups demonstrated p‐c‐Jun+ cells to a lesser extent compared to acute phase but were still in higher frequency compared to SH group. In (A and C), images were cropped from the center of an ROI which reveals stitching lines the result of image tiling and scale bars = 50 µm. In (B), brightness is readjusted for NeuroTrace and scale bar = 10 µm. Deconv., deconvolved image

Immunofluorescently labeled brain tissues evaluated with spinning disk microscope demonstrated NeuN+ cells that were equally dispersed throughout the tissue sections regardless of experimental grouping, with the majority colabeled with NeuroTrace (Figure 2A,B). These cells also colabeled with DRAQ5. Those cells negative for either of NeuroTrace and NeuN, but positive for DRAQ5 were considered glia.

### 3.3 Overview of whole cellular population and data screening

**FIGURE 3:**
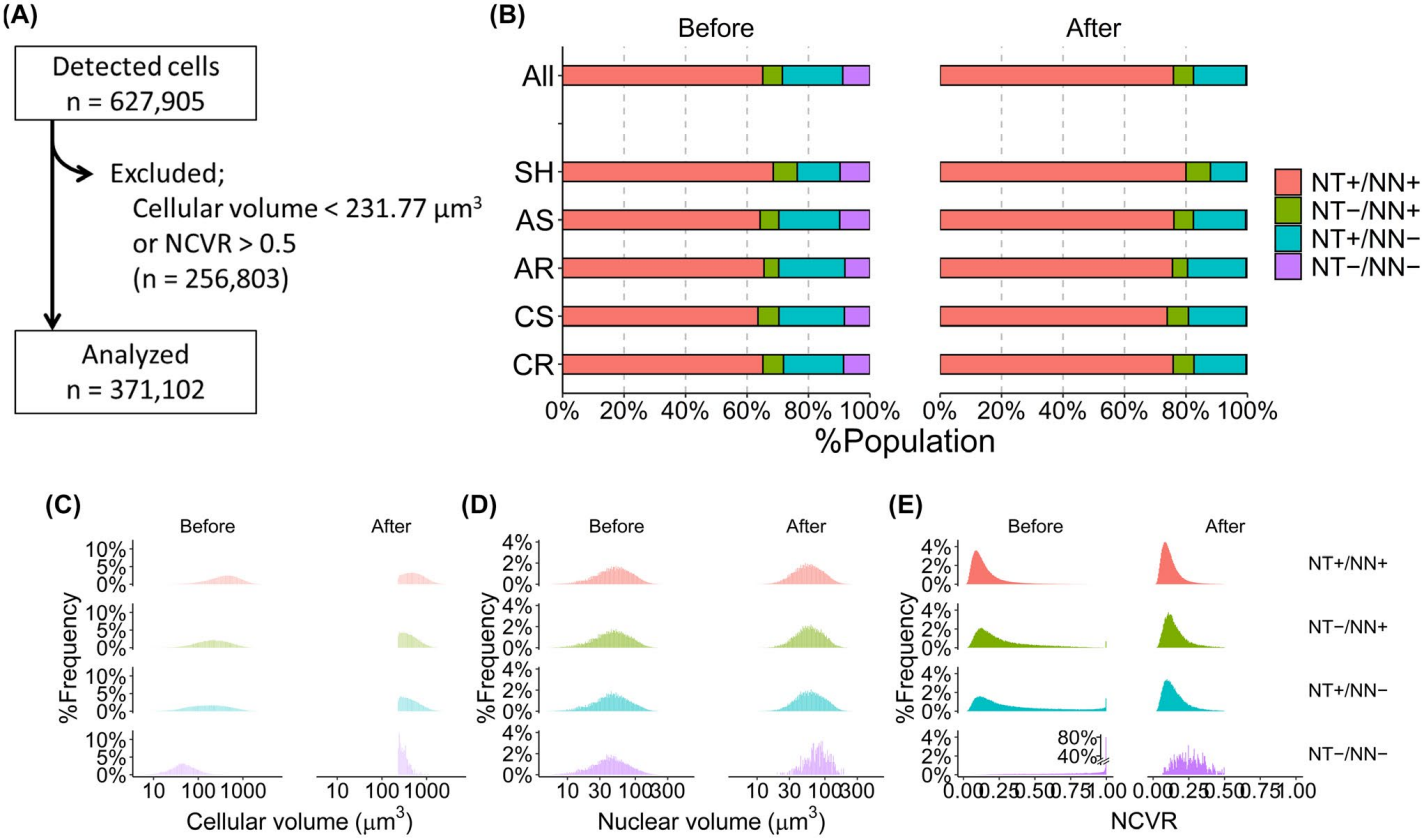
Cell screening strategy to minimize glial contamination. (A) Total 627,905 cells were identified through automated image analysis. Cellular screening based on cellular volume and the volume ratio of nuclear to cell body, NCVR, excluded 256,803 cells with the remaining 371,102 cells subjected to the following assessments. (B–E) Population distributions before and after the screening (left and right of each panel) were depicted for cellular reactivity for NeuroTrace and NeuN (B), cellular volume (C), nuclear volume (D) and NCVR (E), respectively. NCVR, nuclear/cellular volume ratio; NN, NeuN; NT, NeuroTrace

Overall, tissue harvesting proved excellent. Only one tissue section from an animal in the AR group was lost, with only 6 images obtained for this specific animal. Images of ROI placed in somatosensory layer V were automatically processed and 627,905 cells were identified in all the samples evaluated (NeuroTrace+/NeuN+ cells, 400,011 cells [75.6%]; NeuroTrace+/NeuN− cells, 91,778 cells [17.3%]; NeuroTrace−/NeuN+ cells, 34,839 cells [6.6%]; NeuroTrace−/NeuN− cells, 2766 cells [0.5%]) (Figure 3A,B). As described, cell screening was performed using volumetric parameters of individual cells (Figure 3C–E). After cell screening, 371,102 cells remained and were evaluated (Figure 3A). This population included cells positive for both NeuroTrace and NeuN (303,381 cells, 81.8%), those positive for NeuroTrace alone (48,651 cells, 13.1%), those positive for NeuN alone (18,565 cells, 5.0%) and those negative for both (505 cells, 0.1%) (Figure 3B).

### 3.4 Repetitive mild TBI exacerbates the burden of traumatic axonal injury in the acute phase

Employing microscopic examination of the somatosensory neocortex, samples from the AS and AR groups demonstrated neurons with an intense p‐c‐Jun signal distributed primarily in layer V together with a few scattered p‐c‐Jun+ neurons in layer II/III as well as hippocampal dentate gyrus and thalamus. These neurons exhibited a p‐c‐Jun+ profile, that typically equaled or exceeded their nuclear size (Figure 2B,C), a finding previously reported in axotomized neurons at 24hpi [12, 19, 20]. In the SH group, p‐c‐Jun+ profiles were few. In the CS and CR groups, p‐c‐Jun+ profiles were reduced in comparison to the AS or AR groups, but were more frequent than the SH group.

**FIGURE 4:**
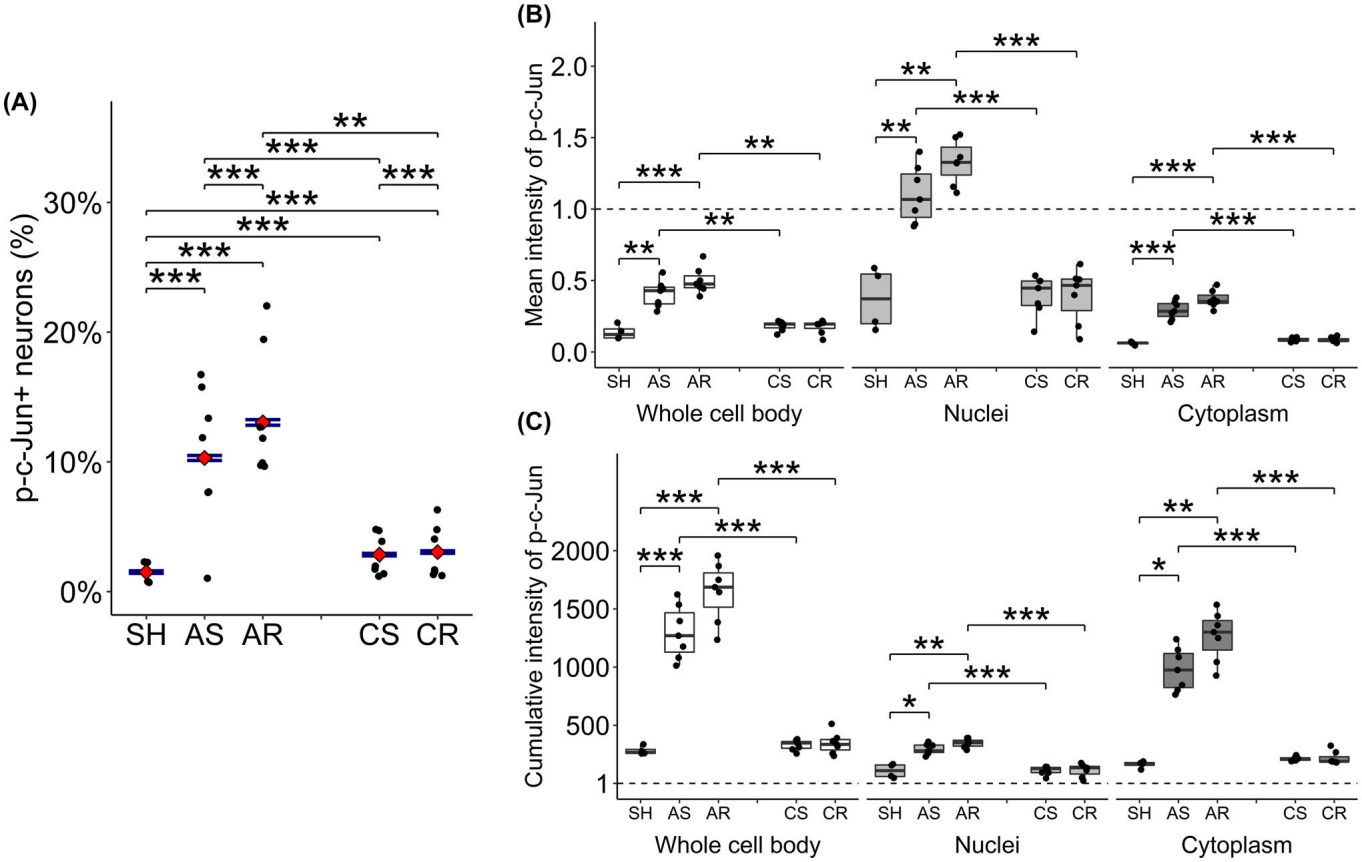
Assessment of p‐c‐Jun expression. (A) Quantitative assessment of p‐c‐Jun+ neurons, presented as a percentage of all NeuroTrace+ neuronal populations. Data are presented as the estimates and 95% confidential interval. At the acute phase of injury, single and repetitive mTBI demonstrated p‐c‐Jun+ expression in 10%–13% of neurons, representing the incidence of TAI. At chronic phase of injury, the percentages decreased to approximately 3%. These values were significantly different in all injured groups from SH groups, between single and repetitive mTBI at the same time points and between acute and chronic time points after the same injury paradigm. (B, C) Assessment of p‐c‐Jun signal intensity. The measurements were performed over the whole cell body (left), within nuclei (center) and within cytoplasm (right). The values were normalized to the threshold value to determine neuronal positivity for p‐c‐Jun. Data are presented as mean ± SEM. (B) Mean signal intensity, representing the concentration of p‐c‐Jun within the corresponding cellular compartment, demonstrated significant increase in all three measurements in AS and AR groups compared to SH and to the corresponding chronic groups. However, only the nuclear measurement exceeded the threshold value, suggesting that the p‐c‐Jun existed in a highly concentrated state within neuronal nuclei relative to their cytoplasm. (C) Cumulative signal intensity, representing the total amount of p‐c‐Jun within the corresponding cellular compartment, demonstrated significant increase in all three measurements in AS and AR groups compared to SH and to the corresponding chronic groups. *p < 0.05, **p < 0.01, ***p < 0.001

The proportion of NeuroTrace+ neurons expressing p‐c‐Jun was quantitatively assessed to evaluate the burden of TAI in both the acute and chronic phase post mTBI (Figure 4A, Table 1 and Table S3).

**TABLE 1:** Quantification of neuronal populations

	SH	AS	AR	CS	CR
NeuroTrace+ cells (/ROI)	1936 ± 105	1691 ± 75	1740 ± 100	2078 ± 46	2120 ± 90
NeuN+ cells (/ROI)	1859 ± 31	1497 ± 44	1486 ± 44	1812 ± 33	1889 ± 35
Fraction of NeuN+ neurons					
in NeuroTrace+ neurons	87.3% (87.1%–87.6%)	81.7% (81.5%–82.0%)	79.9% (79.7%–80.2%)	79.8% (79.5%–80.0%)	81.7% (81.5%–82.0%)
in p‐c‐Jun+ neurons	88.9% (86.8%–90.9%)	89.1% (88.4%–89.7%)	90.7% (90.2%–91.2%)	78.0% (76.5%–79.4%)	78.4% (77.0%–79.7%)
in p‐c‐Jun− neurons	87.3% (87.1%–87.6%)	80.9% (80.6%–81.2%)	78.3% (78.0%–78.6%)	79.8% (79.6%–80.0%)	81.9% (81.6%–82.1%)
p‐c‐Jun+ NeuroTrace+ neurons (/ROI)	29 ± 8	174 ± 31	230 ± 25	59 ± 13	65 ± 17
Fraction of p‐c‐Jun+ neurons in					
NeuroTrace+ neurons	1.50% (1.41%–1.60%)	10.3% (10.1%–10.5%)	10.3% (10.1%–10.5%)	2.85% (2.75%–2.95%)	3.05% (2.95%–3.15%)

Note

Values are presented as “mean ± SEM” for neuronal numbers and “Estimate (95% CI)” for fraction of neuronal populations.

Abbreviations: AR, acute‐phase with repetitive mTBI; AS, acute‐phase with single mTBI; CI, confidential interval; CR, chronic‐phase with repetitive mTBI; CS, chronic‐phase with single mTBI; mTBI, mild traumatic brain injury; ROI, region of interest; SEM, standard error of the means; SH, Sham.

In the acute phase of injury, single and repetitive mTBI demonstrated p‐c‐Jun+ expression in 10%–13% of neurons, representing the incidence of TAI. In the chronic phase of injury, these percentages decreased to approximately 3%. All of these proportions were significantly elevated from the SH group (i.e., SH vs AS, vs AR, vs CS and vs CR). The differences between single and repetitive mTBI at the same time points (i.e., AS vs AR and CS vs CR) and between the acute and the chronic time points after the same injury paradigm (i.e., AS vs CS and AR vs CR) were all significant as well.

Collectively, the results confirmed the increased burden of TAI after single and repetitive mTBI at 24hpi, consistent with our previous study [12]. In contrast, at the more chronic time point, a much smaller proportion of NeuroTrace+ neurons expressed p‐c‐Jun, though that remained significantly elevated relative to SH controls.

### 3.5 Mild TBI induces high concentration of p‐c‐Jun primarily in neuronal nuclei

The mean and cumulative intensities of p‐c‐Jun, representing the concentration and their total amount respectively, were assessed (Figure 4B,C, Table 2, Tables S4 and S5). These measurements were performed over the whole cell body, which included the nuclei and cytoplasmic content. The values were normalized to the threshold value used to differentiate between p‐c‐Jun+ and p‐c‐Jun− subpopulations.

**TABLE 2:** Mean and cumulative signal intensity of p‐c‐Jun

	SH	AS	AR	CS	CR
Mean signal intensity					
Whole cell body	0.140 ± 0.030	0.410 ± 0.040	0.500 ± 0.030	0.180 ± 0.010	0.180 ± 0.020
Nuclei	0.370 ± 0.100	1.10 ± 0.080	1.30 ± 0.060	0.400 ± 0.050	0.400 ± 0.070
Cytoplasm	0.061 ± 0.005	0.290 ± 0.020	0.370 ± 0.020	0.085 ± 0.005	0.084 ± 0.007
Cumulative signal intensity					
Whole cell body	283 ± 18	1300 ± 87	1647 ± 97	330 ± 17	346 ± 35
Nuclei	109 ± 31	295 ± 17	345 ± 14	112 ± 13	113 ± 21
Cytoplasm	163 ± 15	980 ± 69	1265 ± 81	213 ± 7	219 ± 21

Note

Values are presented as “mean ± SEM.” The values are normalized to the threshold value to determine neuronal positivity for p‐c‐Jun.

Abbreviations: AR, acute‐phase with repetitive mTBI; AS, acute‐phase with single mTBI; CR, chronic‐phase with repetitive mTBI; CS, chronic‐phase with single mTBI; mTBI, mild traumatic brain injury; SEM, standard error of the means; SH, Sham.

In both the mean and cumulative intensities, single and repetitive mTBI demonstrated a significantly higher intensity compared to the SH group in the acute phase of injury (i.e., SH vs AS and SH vs AR) followed by significant decrease over time from 24hpi to 28dpi (i.e., AS vs CS and AR vs CR) in all evaluations for each cellular compartment.

### 3.6 Mild TBI does not lead to neuronal loss over 28‐days postinjury

**FIGURE 5:**
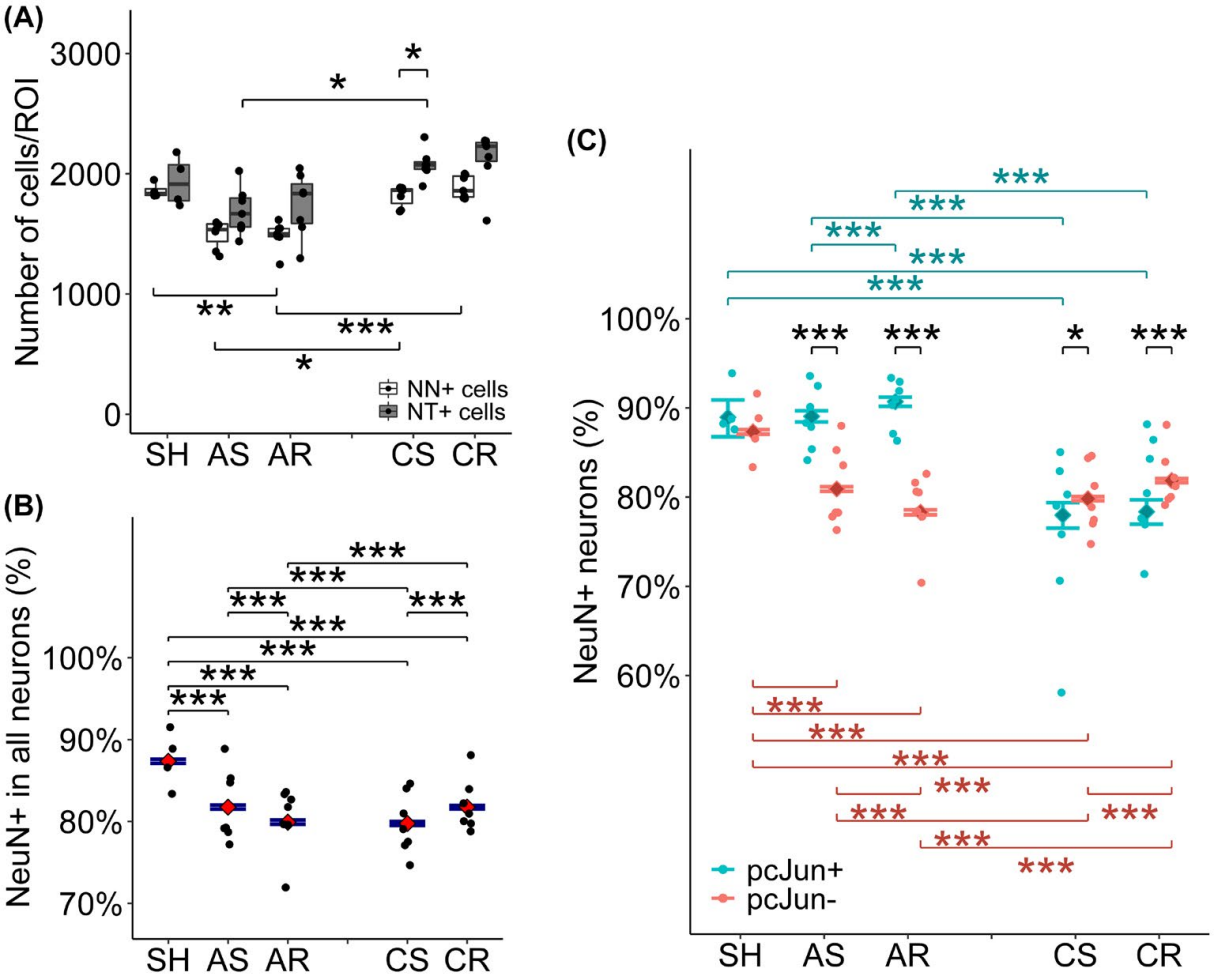
Quantitative assessment of NeuN+ neurons and the marker expression. (A) Absolute numbers of NT+ cells and NeuN+ cells averaged for one ROI. NT+ cells demonstrated no difference from SH group in all groups, suggesting the absence of neuronal loss until 28dpi. In contrast, NeuN+ cells demonstrated significant decrease in the acute phase after repetitive mTBI, followed by returning to SH levels at 28dpi. (B, C) Fraction of NeuN+ cells within NT+ neurons (B) and within p‐c‐Jun+ and p‐c‐Jun− neurons (C). (B) NeuN+ cells accounted for 87% of neurons in SH group. In contrast, the other groups delineated 80%, significantly lower from SH group. A significant difference was observed between single and repetitive mTBI at both time points and between acute and chronic phase regardless of number of injuries. (C) NeuN+ neurons accounted for 87%–90% of either populations of SH group and p‐c‐Jun+ neurons at acute phase of injury, significantly higher than p‐c‐Jun− neurons. In contrast, at chronic phase, only 78% of p‐c‐Jun+ neurons were NeuN+ while the fraction increased to 80%–82% in p‐c‐Jun− neurons. (A): mean ± SEM, (B, C): estimates and 95% confidential interval. *p < 0.05, **p < 0.01, ***p < 0.001; NN, NeuN; NT, NeuroTrace

The total neuronal population in the neocortex assessed was evaluated using NeuN together with NeuroTrace (Figure 5A, Table 1, Table S6 and S7).

NeuroTrace+ cells demonstrated no differences in both the acute and chronic phases of injury compared to the SH group, regardless of either single or repetitive injury. In contrast, repetitive mTBI significantly reduced the number of NeuN+ cells relative to the SH group in the acute phase (i.e., in AR group). Both single and repetitive mTBI elicited a significant increase in NeuN+ cellular numbers over time from 24hpi to 28dpi, returning to the SH levels. Within the neuronal population, defined by its positivity for NeuroTrace, the total NeuN+ neurons accounted for 87% of NeuroTrace+ neurons in the SH group, while delineating 80% in the other groups (Figure 5B, Table S7). These proportions in each injured group were all significantly lower than that observed in the SH group respectively, suggesting no neuronal loss but rather an attenuated NeuN expression.

### 3.7 Attenuated NeuN expression does not correspond to p‐c‐Jun expression in the acute phase but does so at the later time point

The expression of NeuN was further investigated for individual p‐c‐Jun+ and p‐c‐Jun− neurons (Figure 5C, Table S7). Approximately 87%–91% of p‐c‐Jun+ neurons were NeuN positive in the SH group. After mTBI, this fraction remained at as the same level as p‐c‐Jun+ neurons in the acute phase. In contrast, it significantly decreased and approached 80% or below in the p‐c‐Jun− neurons irrespective of the number of injuries. In the chronic phase, the fraction of p‐c‐Jun+ neurons dropped significantly to 78%, significantly lower than the p‐c‐Jun− neurons, which remained at 80%–82%.

### 3.8 Neurons expressing p‐c‐Jun exhibit higher amounts of NeuN accumulation in the acute phase, but its concentration remains unchanged

**FIGURE 6:**
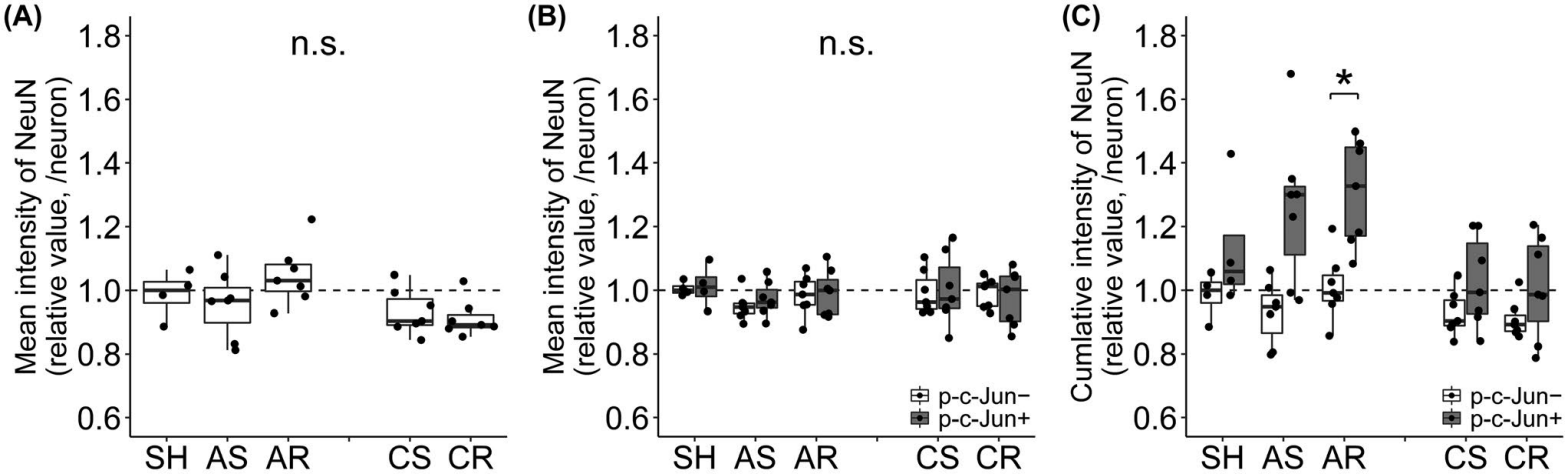
Assessment of NeuN signal intensity. (A, B) The mean signal intensity, representing NeuN concentration, assessed for whole neuronal population (A) and for p‐c‐Jun+ and p‐c‐Jun− neurons (B). There was no difference in all comparisons. (C) The cumulative signal intensity, representing total amount of NeuN, assessed for p‐c‐Jun+ and p‐c‐Jun− neurons. P‐c‐Jun+ neurons demonstrated significantly higher value compared to p‐c‐Jun− neurons in AR group. Either neuronal population did not demonstrate inter‐group difference. Data are presented as mean ± SEM. The values were averaged for single neurons then normalized to the median of SH group (A) or normalized to the median of p‐c‐Jun− neurons in SH group (B, C). *p < 0.05, **p < 0.01, ***p < 0.001

The mean signal intensity of NeuN expression, representing the concentration of NeuN, within the whole neuronal population was assessed (Figure 6A, Table 3 and Table S8). The values were averaged for single neurons and normalized to the median of the SH group. No significant difference was demonstrated in all inter‐group comparisons.

**TABLE 3:** Mean and cumulative signal intensity of NeuN

	SH	AS	AR	CS	CR
Mean signal intensity					
Whole neuronal population	0.988 ± 0.0376	0.958 ± 0.0403	1.05 ± 0.0357	0.932 ± 0.0266	0.912 ± 0.0219
p‐c‐Jun+ neurons	1.00 ± 0.047	0.968 ± 0.018	0.993 ± 0.028	1.00 ± 0.049	0.987 ± 0.035
p‐c‐Jun− neurons	1.01 ± 0.015	0.949 ± 0.017	0.988 ± 0.026	0.991 ± 0.028	0.990 ± 0.018
Cumulative signal intensity					
p‐c‐Jun+ neurons	1.12 ± 0.103	1.25 ± 0.095	1.29 ± 0.059	1.07 ± 0.059	1.02 ± 0.069
p‐c‐Jun− neurons	0.989 ± 0.041	0.949 ± 0.040	1.04 ± 0.041	0.942 ± 0.029	0.917 ± 0.023

Note

Values are presented as “mean ± SEM.” “Whole neuronal population”; values are averaged for single neurons and normalized to the median of SH group. “p‐c‐Jun+ neurons” and “p‐c‐Jun‐ neurons”; values are averaged for single neurons and normalized to the median of p‐c‐Jun‐ neurons in SH group.

Abbreviations: AR, acute‐phase with repetitive mTBI; AS, acute‐phase with single mTBI; CR, chronic‐phase with repetitive mTBI; CS, chronic‐phase with single mTBI; mTBI, mild traumatic brain injury; SEM, standard error of the means; SH, Sham.

Next, the mean and cumulative intensity of NeuN, representing the concentration and the total amount of NeuN respectively, were further assessed in the p‐c‐Jun+ and p‐c‐Jun− neurons. These values were normalized to the median of p‐c‐Jun− neuronal populations in the SH group.

In the mean signal intensity (Figure 6B, Table 3 and Table S8), no significant difference was demonstrated in all inter‐group comparisons. In the cumulative intensity (Figure 6C, Table 3 and Table S9), repetitive mTBI demonstrated significantly higher value in p‐c‐Jun+ neurons compared to p‐c‐Jun− neurons in the acute phase. However, neither of p‐c‐Jun+ and p‐c‐Jun− neurons demonstrated significant difference in the inter‐group comparisons.

### 3.9 Neurons expressing p‐c‐Jun exhibit cellular/neuronal hypertrophy in the acute phase while those neurons losing NeuN expression exhibit cellular/neuronal atrophy

**FIGURE 7:**
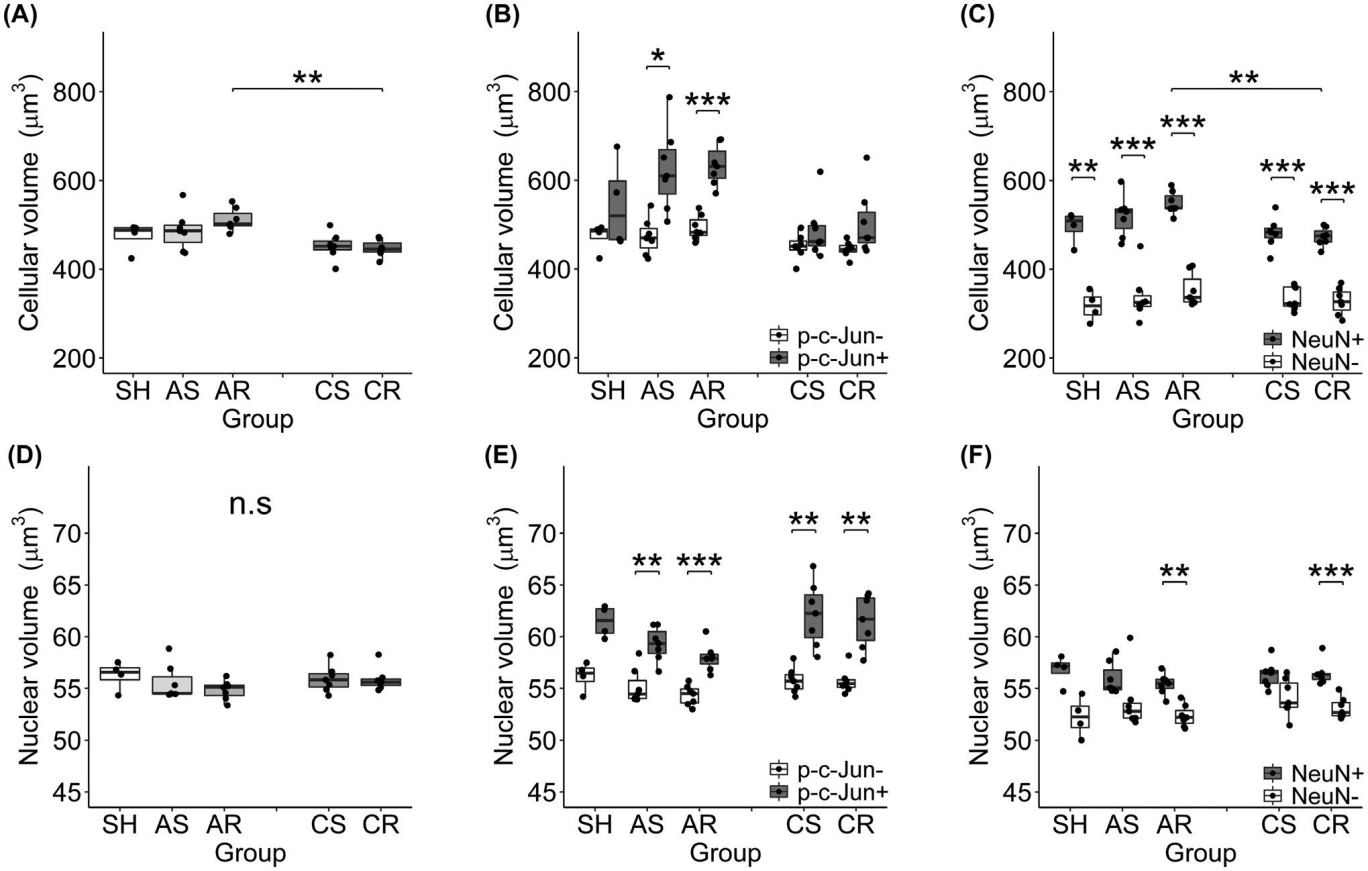
(A–F) Morphological assessment of neurons. Data are presented as mean ± SEM. Cellular volume (upper row) and nuclear volume (lower row) were assessed for the entire neuronal population (A, D), p‐c‐Jun+ and p‐c‐Jun− neuronal populations (B, E) and NeuN+ and NeuN− neuronal populations (C, F). (A, D) In assessment for entire neuronal populations, none of the groups demonstrated significant difference compared to SH group in either cellular or nuclear volumes. Repetitive mTBI demonstrated significant decrease in the cellular volume from 24hpi to 28dpi. (B, E) In assessment for p‐c‐Jun+ and p‐c‐Jun− neuronal populations, none demonstrated significant change compared to corresponding neurons in SH group in either cellular or nuclear volumes. However, p‐c‐Jun+ neurons demonstrated significantly larger cellular and nuclear volume compared to p‐c‐Jun− neurons at acute phase. P‐c‐Jun+ neurons revealed larger nuclear volume at chronic time point as well, but cellular volume was not different. (C, F) In assessment for NeuN+ and NeuN− neuronal populations, none demonstrated significant change compared to corresponding neurons in SH group in either cellular or nuclear volumes. However, NeuN+ neurons consistently demonstrated significantly larger cellular volumes compared to NeuN− neurons in all groups. NeuN+ neurons revealed significantly larger nuclear volume compared to NeuN− neurons only in AR and CR groups. *p < 0.05, **p < 0.01, ***p < 0.001

To evaluate morphological change related to the burden of mTBI, the cellular and nuclear volumes were assessed first for whole neuronal population of each experimental group (Figure 7A,D, Table 4, Tables S10 and S11). In the acute phase of injury, neither of the cellular and nuclear volumes demonstrated difference in any of the injured groups compared to the SH group. In terms of the change over time, the cellular volume demonstrated significant decrease from 24hpi to 28dpi after repetitive mTBI (i.e., AR vs CR) (Figure 7A, Table S10).

**TABLE 4:** Cellular and nuclear volume of neurons by injury mode

	SH	AS	AR	CS	CR
Cellular volume of neurons (μm^3^)					
Whole neuronal population	474 ± 17	487 ± 17	512 ± 10	452 ± 11	447 ± 7
p‐c‐Jun+ neurons	545 ± 51	626 ± 36	634 ± 17	487 ± 24	506 ± 28
p‐c‐Jun− neurons	473 ± 16	474 ± 16	493 ± 11	451 ± 11	445 ± 7
NeuN+ neurons	496 ± 18	520 ± 18	550 ± 10	482 ± 13	473 ± 8
NeuN− neurons	317 ± 17	338 ± 21	353 ± 14	335 ± 10	328 ± 12
Nuclear volume of neurons (μm^3^)					
Whole neuronal population	56 ± 0.7	56 ± 0.6	55 ± 0.4	56 ± 0.5	56 ± 0.4
p‐c‐Jun+ neurons	61 ± 0.8	59 ± 0.6	58 ± 0.5	62 ± 1	61 ± 1
p‐c‐Jun− neurons	56 ± 0.7	55 ± 0.6	54 ± 0.4	56 ± 0.5	56 ± 0.5
NeuN+ neurons	57 ± 0.7	56 ± 0.6	55 ± 0.4	56 ± 0.5	56 ± 0.4
NeuN− neurons	52 ± 1	54 ± 1	52 ± 0.4	54 ± 0.7	53 ± 0.4

Note

Values are presented as “mean ± SEM (µm^3^).”

Abbreviations: AR, acute‐phase with repetitive mTBI; AS, acute‐phase with single mTBI; CR, chronic‐phase with repetitive mTBI; CS, chronic‐phase with single mTBI; mTBI, mild traumatic brain injury; SEM, standard error of the means; SH, Sham.

Next, both the cellular and nuclear volumes were further evaluated in relation to p‐c‐Jun positivity to determine if neuronal p‐c‐Jun expression parallels morphological change (Figure 7B,E, Table 4, Tables S10 and S11). There were no differences in either of the cellular and nuclear volumes in any of the injured groups compared to the SH group irrespective of p‐c‐Jun positivity. However, p‐c‐Jun+ neurons demonstrated significantly larger cellular and nuclear volumes compared to p‐c‐Jun− neurons in the acute phase. Those positive for p‐c‐Jun revealed larger nuclear volumes at the chronic time point as well.

The same parameters were also evaluated in relation to NeuN positivity to investigate if attenuation of NeuN reactivity coincides with any morphological change (Figure 7C,F, Table 4, Tables S10 and S11). There was no difference in either of the cellular and nuclear volumes in any of the injured groups compared to the SH group irrespective of NeuN positivity. However, NeuN+ neurons consistently demonstrated significantly larger cellular volume compared to NeuN− neurons in all groups. NeuN+ neurons revealed significantly larger nuclear volume compared to NeuN− neurons only in the AR and CR groups.

### 3.10 Larger neurons are at a greater risk of axotomy while smaller neurons demonstrate an increased chance of NeuN expression loss

The potential relationship between the cellular volume and the occurrence of axotomy or the attenuated NeuN expression were evaluated, dividing NeuroTrace+ neurons into quintiles based on cellular volume.

**FIGURE 8:**
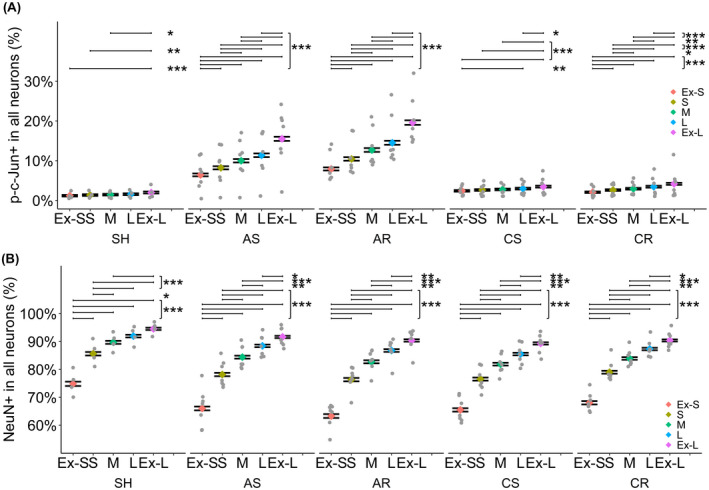
Fraction of p‐c‐Jun+ or NeuN+ neurons dependent on cellular volume. NeuroTrace+ neurons were divided into the quintiles based on cellular volume in each animal (Ex‐S, S, M, L, Ex‐L), then the fraction of p‐c‐Jun+ neurons or NeuN+ neurons were estimated. Data are presented as the estimates and 95% confidential interval. (A) Fraction of p‐c‐Jun+ neurons and cellular volume. Larger neurons consistently demonstrated higher percentage of p‐c‐Jun+ neurons regardless of the number of injuries and time points. The majority of the paired comparisons between the quintiles within each experimental group demonstrated significant differences. (B) Fraction of NeuN+ neurons and cellular volume. There was a consistent trend that larger neurons were more likely to express NeuN than smaller neurons, in other words, smaller neurons have an increased incidence of NeuN expression loss. All pair comparisons between the quintiles within each experimental group demonstrated significant differences, with an exception for two pair comparisons (M vs L and L vs Ex‐L in SH group). Ex‐L, extra‐large; Ex‐S, extra‐small; L, large; M, medium; S, small. *p < 0.05, **p < 0.01, ***p < 0.001

In terms of axotomy, larger neurons consistently demonstrated higher percentage of p‐c‐Jun+ neurons regardless of the number of injuries and time points (Figure 8A, Table 5 and Table S12). The majority of the paired comparisons between the quintiles within each experimental group demonstrated significant differences. In terms of NeuN expression within the same quintiles based upon the cellular volume (Figure 8B, Table 5 and Table S13), there was a consistent trend that larger neurons were more likely to express NeuN than smaller neurons, in other words, smaller neurons have an increased incidence of NeuN expression loss. All pair comparisons between the quintiles within each experimental group demonstrated significant differences, with an exception for two pair comparisons (M vs L and L vs Ex‐L in the SH group).

**TABLE 5:** Fraction of p‐c‐Jun+ neurons and NeuN+ neurons depending on cellular volume

	SH	AS	AR	CS	CR
p‐c‐Jun+ neuronal fraction					
Ex‐S	1.19% (1.00%–1.39%)	6.43% (6.08%–6.79%)	7.93% (7.55%–8.32%)	2.41% (2.21%–2.61%)	2.07% (1.89%–2.26%)
S	1.35% (1.15%–1.57%)	8.22% (7.83%–8.62%)	10.4% (10.0%–10.9%)	2.63% (2.42%–2.84%)	2.61% (2.41%–2.82%)
M	1.44% (1.24%–1.67%)	9.99% (9.56%–10.4%)	12.7% (12.2%–13.2%)	2.77% (2.56%–2.99%)	2.94% (2.73%–3.16%)
L	1.56% (1.35%–1.79%)	11.4% (10.9%–11.8%)	14.5% (14.0%–15.0%)	2.97% (2.76%–3.20%)	3.45% (3.23%–3.69%)
Ex‐L	1.98% (1.75%–2.25%)	15.5% (15.0%–16.0%)	19.6% (19.0%–20.2%)	3.47% (3.24%–3.71%)	4.18% (3.92%–4.44%)
NeuN+ neuronal fraction					
Ex‐S	74.9% (74.1%–75.6%)	66.0% (65.3%–66.7%)	63.3% (62.6%–64.0%)	65.5% (64.9%–66.1%)	68.1% (67.5%–68.6%)
S	85.6% (85.0%–86.2%)	78.2% (77.6%–78.8%)	76.3% (75.7%–77.0%)	76.5% (76.0%–77.1%)	79.0% (78.5%–79.5%)
M	89.7% (89.2%–90.3%)	84.4% (83.9%–84.9%)	82.7% (82.2%–83.3%)	81.9% (81.4%–82.4%)	83.9% (83.5%–84.4%)
L	92.0% (91.5%–92.4%)	88.4% (88.0%–88.9%)	86.8% (86.3%–87.3%)	85.5% (85.1%–86.0%)	87.3% (86.9%–87.8%)
Ex‐L	94.6% (94.1%–95.0%)	91.7% (91.3%–92.0%)	90.4% (90.0%–90.8%)	89.3% (88.9%–89.7%)	90.4% (90.0%–90.8%)

Note

Values are presented as “Estimate (95% CI).”

Abbreviations: AR, acute‐phase with repetitive mTBI; AS, acute‐phase with single mTBI; CI, confidential interval; CR, chronic‐phase with repetitive mTBI; CS, chronic‐phase with single mTBI; Ex‐L, extra‐large; Ex‐S, extra‐small; L, large; M, middle; mTBI, mild traumatic brain injury; OR, odds ratio; ROI, region of interest; S, small; SEM, standard error of the means; SH, Sham.

## 4 DISCUSSION

The current study focused on the acute and chronic consequences of mTBI and their exacerbation by repetitive injury. To that end, the potential for neuronal cell death was evaluated and placed in the context of axotomy. The results of this study confirm that repetitive mTBI increases the burden of axotomy, as shown by an increased proportion of NeuroTrace+ neurons expressing p‐c‐Jun, a somatic marker of axotomy, in the acute phase of injury. Importantly, this proportional increase in neurons expressing p‐c‐Jun persisted, although this level of expression diminished greatly at 28dpi. Further, repetitive injury and the subsequent increased burden of axotomy did not result in neuronal death, with no significant effect of the injury upon the NeuroTrace+ neuronal number. Collectively, repetitive mTBI exacerbates the burden of axotomy, with these effects persisting in a subpopulation of neurons at 28dpi. The occurrence of widespread axotomy in large fields of intact neurons does not support the premise that repetitive mTBI elicits progressive cell death as a major cause of morbidity. Rather, this finding speaks to the occurrence of an enduring, axotomy‐mediated circuit disruption.

The transcription factor c‐Jun is one of the substrates of c‐Jun N‐terminal kinases (JNKs) and is an indispensable bifunctional molecule in neurons involved in processes leading to neuronal cell death or alternatively neuroprotective functions such as neurite outgrowth and neuronal regeneration [38, 39, 40, 41, 42]. After axonal transection, the chronic activation of JNK concomitant with c‐Jun phosphorylation, associated with neuronal regeneration, has been described [43, 44]. In the same cFPI model, a tight linkage between axotomized neurons and their expression of p‐c‐Jun exists together with the concomitant activation of activating transcription factor 3 (ATF3), another transcription factor linked to axon regeneration [19]. In this previous study, 85%–95% of axotomized pyramidal neurons in the cortical layer V expressed p‐c‐Jun after diffuse mTBI, in contrast to non‐axotomized neurons of which less than 3% did so [19]. Another study investigating interneuronal populations in the same cortical area demonstrated 64% sensitivity and 98% specificity of p‐c‐Jun as a marker to identify those undergoing axotomy [20]. In the current study, more than 10% of the total neuronal population revealed axonal injury at 24hpi. This proportion was higher after repetitive mTBI in comparison to a single mTBI, corroborating our previous study demonstrating that repetitive mTBI exacerbates the number of axotomized neurons [12]. The p‐c‐Jun+ neurons observed in the acute‐injured groups demonstrated increased mean nuclear p‐c‐Jun signal intensity, representing a highly concentrated p‐c‐Jun within this cellular compartment, which was paralleled by somatic hypertrophy. This acute phase somatic hypertrophy in axotomized neurons is consistent with the well‐known neuronal response to axonal damage termed “axon reaction,” wherein neurons display swelling with dislocation and movement of fragmented rough endoplasmic reticulum toward the periphery [45, 46]. Accordingly, the current observations confirm the utility and validity of p‐c‐Jun as a marker of axotomized neurons in the acute phase of injury. Of further note, are our findings that the proportions of NeuroTrace+ neurons expressing p‐c‐Jun decreased at the more chronic time point, though these proportions were still significantly elevated over the level found in the SH group in the absence of neuronal death. Although unexpected, this observation at the chosen chronic time point may represent another biological component of the p‐c‐Jun response in which the phosphorylation of c‐Jun in axotomized neurons has also been implicated in neuronal regeneration and plasticity [41, 47]. Specifically, it has been posited that its downregulation occurs only after successful regeneration or alternatively neuronal quiescence or death [48, 49]. Injured cortical neurons have been demonstrated to sprout and mount a regenerative attempt following neuronal injuries including diffuse mTBI [19], laser‐mediated single neuron axotomy [50] and focal TBI [51, 52, 53]. The regeneration occurring with cortical neuronal axotomy has been associated with the subsequent ectopic formation of synaptic boutons [50]. Thus, the early expression of p‐c‐Jun after mTBI with its subsequent reduction at 28dpi without neuronal death suggests the potential for ectopic axon reorganization/repair that may in turn contribute to chronic network dysfunction. In concert with these findings, we also observed that larger neurons appeared more likely to be axotomized. One possible explanation is that neurons with varying size are differently vulnerable to the injury. Specifically, the larger neurons are more likely to be projection neurons with longer trajectories into the white matter, where they are more widely exposed to the forces of injury. It is also possible that the hypertrophic somatic change occurring in axotomized neurons may inflate the average cellular volume of axotomized neuronal subgroup, resulting in relatively larger volumes compared to those non‐axotomized. However, this hypothesis seems technically difficult to verify in vivo, as it require fate of single neuron to be monitored from the time of injury.

Another consideration derived from the current observations calls into question the use of NeuN, a common immunohistochemical marker of neurons, in quantitative assessments of neuronal numbers. Specifically, our studies demonstrate that the loss of NeuN+ neurons may not reflect actual neuronal loss but rather an attenuated neuronal NeuN expression. In the current study, NeuroTrace+ neuronal numbers revealed no reduction at either 24hpi or 28dpi compared to SH group, providing no evidence of any injury‐induced neuronal loss. In contrast, a decreased NeuN+ neuronal population was found at 24hpi after repetitive mTBI in parallel with a significantly reduced proportion of NeuroTrace+ neurons expressing NeuN compared to the SH group. In the absence of parallel NeuroTrace+ neuronal quantification, the loss of NeuN+ neurons has been routinely interpreted as neuronal loss. However, our results demonstrate otherwise, mandating caution when using NeuN to assess neuronal viability or loss following brain injury. This issue has been recently raised in another TBI model [54] as well as in other experimental paradigms in both CNS and PNS [24, 55, 56, 57, 58, 59, 60, 61]. Adding further complexity to our understanding of NeuN’s role in neuronal viability are reports that alterations of NeuN expression show differences in the CNS vs the PNS. Some have reported no altered NeuN expression in axotomized CNS neurons, in contrast to axotomized PNS neurons in which NeuN expression is altered [24, 55, 59, 61]. In our study, a reduced proportion of NeuN+ neurons was observed only in non‐axotomized neurons in the acute phase of injury. In contrast, in axotomized neurons, the decreased proportion of NeuN+ neurons was only demonstrated at the chosen chronic survival time, suggesting a delayed response to the injury leading to the attenuated NeuN expression in axotomized neurons compared to those non‐axotomized. This finding is consistent with a recent study demonstrating a loss of detectable NeuN immunoreactivity associated with p‐c‐Jun expressing axotomized bulbospinal neurons at 30‐days postinjury [57]. In terms of the NeuN expression level in single neurons, the expression level increased in axotomized neurons acutely but did not persist, reverting to the level of the sham‐injured animals by 28dpi, in contrast to the unchanged level in non‐axotomized neurons. Such different responses to the burden of mTBI suggest the different mechanisms for axotomized versus non‐axotomized neurons leading to the attenuation of NeuN expression. As noted, small neurons were more likely to lose their NeuN expression. Importantly, NeuN, also known as a pre‐mRNA alternative splicing regulator RbFox3 [62], was recently demonstrated to play a role in neuronal development and plasticity, including hippocampal neurogenesis and synaptogenesis [63], the maintenance of hippocampal circuitry balance and function [64] and the maturation and the assembly of axon initial segment [65], potentially leading to a subsequent neuronal circuit dysfunction. Although the extent to which altered expression of NeuN/RbFox3 following axotomy may affect plasticity remains to be determined, the potential for a divergent response within axotomized and non‐axotomized neurons involving NeuN expression mandates future study.

In human mTBI, recent clinical studies using conventional magnetic resonance imaging (MRI) suggest that transient cortical thickening occurs acutely, followed by subsequent thinning of the same area with global brain atrophy a year later [66, 67, 68, 69, 70, 71, 72]. Unfortunately, conventional imaging modalities such as computed tomography (CT) and conventional MRI are not capable of recognizing mTBI‐related microscopic change occurring early after insult. An advanced MRI modality, diffusion tensor imaging (DTI), can visualize TAI‐related microscopic changes and is thought to be promising tool to assess the burden of TAI/DAI and predict outcome. However, to date, its full utility has not been established in mTBI cases [73, 74, 75, 76, 77] and DTI is not capable of detecting the underlying pathology at either the cellular or subcellular level. In patients, it is impossible to histologically investigate how acute phase mTBI‐related microscopic pathology transitions to macroscopic structural change detectable by this clinical imaging modality. Thus, at present, animal models of TBI remain an invaluable tool for unveiling mechanisms underlying the progress of mTBI‐related pathology.

The current communication's focus on axotomy and the potential for cell death must be considered of added significance in the context of the spectrum of TBI severity and its relevance to milder forms of TBI and/or injuries of comparable severity that are followed by repetitive insults. Many have reported multiple forms of cell injury and death following mTBI with descriptions of necrosis, autophagy, apoptosis and pyroptosis [78, 79, 80, 81, 82]. Typically, in these descriptive studies, only one or two markers of injury and death were employed, with most assessed with the confines of more severe pathological conditions such as a contusion or its related pericontusional domains. Moreover, the expression of these markers appeared dependent on the severity of injury and its repetition, with their numbers increasing with injury intensity and the intervals between repetitive insults. Thus, although limited and focal neuronal cell death has been described following TBI, with many advocating specific cell pathways leading to their demise, it is important to note that these scattered forms of neuronal cell death provide a very incomplete appreciation of those cellular changes occurring postinjury. Importantly, our large‐scale, cell‐based neocortical evaluations in mTBI supports very rare occurrence of cell death and demonstrate a marked and divergent alteration in NeuN expression within axotomized and non‐axotomized neurons following diffuse brain injury. Accordingly, our results call for a reassessment of neuronal perturbation in the milder forms of injury, arguing that more subtle forms of structural and functional changes are at work in exerting subsequent morbidity.

Although somewhat peripheral to the focus of the current investigation, it is important to note that the overall burden of axotomy following injury can be influenced by brain/body temperature, with several previous studies demonstrating a hyperthermia‐induced exacerbation of the axonal damage as well as its associated cognitive function and inflammatory responses [83, 84, 85]. In contrast, hypothermia has been shown to exert axonal protection [86, 87, 88, 89]. Because of these findings the current study was conducted under pretraumatic normothermic conditions. Although we cannot preclude the occurrence of modest posttraumatic hypothermia in the sham and injured animals, this in itself, would have no role in exacerbating the axonal response to injury.

Because the current study supports the premise that neither singular nor repetitive mTBI results in neuronal death, it appears most likely that the increasing burden of axonal injury associated with repetitive brain injury must be a player in any subsequent morbidity. Based upon our previous reports that a singular mTBI evokes deafferentation and neuroplastic change as well as electrophysiological abnormalities within the neocortex [21], there is the need to assess these specific changes following repetitive injury over a relatively elongated survival period. Only then will we understand how CNS circuits are injured and how these circuits reorganize postinjury and subsequently influence behavioral recovery.

## 5 CONCLUSIONS

We demonstrate an increased burden of TAI after repetitive mTBI, which is most striking in the acute phase response of injury. Our finding of widespread axotomy in large fields of intact neurons rejects the premise that repetitive mTBI elicits progressive neuronal death, rather emphasizing the importance of axotomy‐mediated change.

## CONFLICT OF INTEREST

The authors declare that they have no conflict of interest.

## AUTHOR CONTRIBUTIONS

Yasuaki Ogino, Tytus Bernas and John T. Povlishock contributed to the study conceptualization and design. Yasuaki Ogino contributed to conducting experiments, sample collection and data acquisition. Tytus Bernas contributed to building automated image analyzing algorithm and performing image analysis. Yasuaki Ogino contributed to statistical analysis, visualizing data and wrote the first draft of the manuscript. All authors contributed to interpretation of the results, revised the first draft, read and approved the final manuscript.

## ETHICS APPROVAL

Experimental protocols conducted in this study were approved by Institutional Animal Care and Use Committee of Virginia Commonwealth University.

## CLINICAL TRIAL REGISTRATION

Not applicable.

## PATIENT CONSENT STATEMENT

Not applicable.

## PERMISSION TO REPRODUCE MATERIAL FROM OTHER SOURCES

Not applicable.

## Supporting information

Fig S1
**Figure S1** A scatterplot of maximum signal intensity of NeuN and NeuroTrace. Dashed lines indicate threshold values to determine the positivity for each marker. These values were empirically determined by two expert observers using randomly selected images from the experimental image set. Note that both markers demonstrated homogeneity in maximum signal intensity distribution. Both X and Y axes are shown in logarithmic scale

Table S1‐S13
**Table S1** Full list of R package used in this work
**Table S2** Physiological data, injury intensity and LORR
**Table S3** Quantification of p‐c‐Jun+ neurons
**Table S4** Mean signal intensity of p‐c‐Jun
**Table S5** Cumulative signal intensity of p‐c‐Jun
**Table S6** Quantification of NeuroTrace+ cells and NeuN+ cells
**Table S7** Fraction of NeuN+ neurons
**Table S8** Mean signal intensity of NeuN
**Table S9** Cumulative signal intensity of NeuN
**Table S10** Cellular volume of neurons by injury mode
**Table S11** Nuclear volume of neurons by injury mode
**Table S12** Fraction of p‐c‐Jun+ neurons depending on cellular volume
**Table S13** Fraction of NeuN+ neurons depending on cellular volume

Supplementary Material

## Data Availability

Data available on request from the authors.
