# Corrigendum

**DOI:** 10.1111/bpa.13103

**Published:** 2022-06-26

In the article by Ogino et al.,^1^ the following corrections are required:

- In Table 1, the fraction of p‐c‐Jun+neurons in NeuroTrace+ neurons for AR group (Estimate and 95%CI) is shown as “10.3% (10.1–10.5%).” This should be “13.0% (12.8–13.3%).” The corrected Table 1 is shown below:
  <New Table 1 here>
- In Table S3, the fraction of p‐c‐Jun+neuronal population for AR group (Estimate and 95%CI) is shown as “10.3% (10.1–10.5%).” This should be “13.0% (12.8–13.3%).” Corrected version of Table S3 is corrected online.
