# Supplementary figures and images for: Axonal injury following mild traumatic brain injury is exacerbated by repetitive insult and is linked to the delayed attenuation of NeuN expression without concomitant neuronal death in the mouse
Source: Brain Pathol. 2021 Nov 3;32(2):e13034. doi: 10.1111/bpa.13034 (PMC8877729; doi:10.1111/bpa.13034)

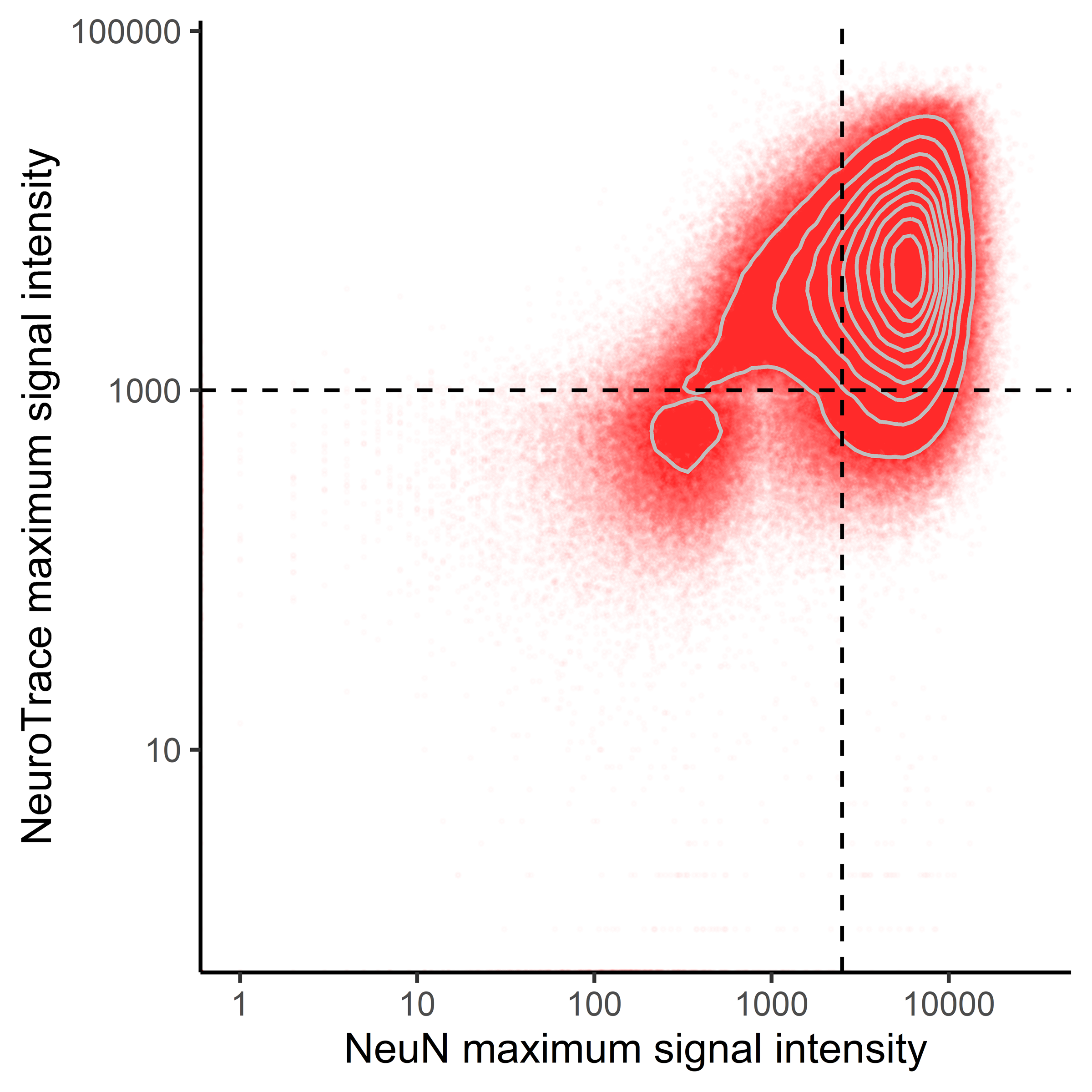

Supplement: Supplementary file 1 — Fig S1 Figure S1 A scatterplot of maximum signal intensity of NeuN and NeuroTrace. Dashed lines indicate threshold values to determine the positivity for each marker. These values were empirically determined by two expert observers using randomly selected images from the experimental image set. Note that both markers demonstrated homogeneity in maximum signal intensity distribution. Both X and Y axes are shown in logarithmic scale [file BPA-32-e13034-s002.tif]
