# Supplementary material for: Axonal injury following mild traumatic brain injury is exacerbated by repetitive insult and is linked to the delayed attenuation of NeuN expression without concomitant neuronal death in the mouse
Source: Brain Pathol. 2021 Nov 3;32(2):e13034. doi: 10.1111/bpa.13034 (PMC8877729; doi:10.1111/bpa.13034)
Supplement: Supplementary file 2 — Table S1‐S13 Table S1 Full list of R package used in this work Table S2 Physiological data, injury intensity and LORR Table S3 Quantification of p‐c‐Jun+ neurons Table S4 Mean signal intensity of p‐c‐Jun Table S5 Cumulative signal intensity of p‐c‐Jun Table S6 Quantification of NeuroTrace+ cells and NeuN+ cells Table S7 Fraction of NeuN+ neurons Table S8 Mean signal intensity of NeuN Table S9 Cumulative signal intensity of NeuN Table S10 Cellular volume of neurons by injury mode Table S11 Nuclear volume of neurons by injury mode Table S12 Fraction of p‐c‐Jun+ neurons depending on cellular volume Table S13 Fraction of NeuN+ neurons depending on cellular volume [file BPA-32-e13034-s003.docx]

Supplementary Table 1; Full list of R package used in this work

| **Package name** | **Version** | **Maintainer** | **References** |
| --- | --- | --- | --- |
| brunnermunzel | 1.4.1 | Toshiaki Ara | Ara T (2020) Brunnermunzel: (Permuted) brunner-munzel test |
| car | 3.0.10 | John Fox | Fox J, Weisberg S (2019) An R companion to applied regression, Third. Sage, Thousand Oaks CA |
| carData | 3.0.3 | John Fox | Fox J, Weisberg S, Price B (2019) CarData: Companion to applied regression data sets |
| compute.es | 0.2.4 | AC Del Re | Re ACD (2013) Compute.es: Compute effect sizes |
| exactRankTests | 0.8.31 | Torsten Hothorn | Hothorn T, Hornik K (2019) ExactRankTests: Exact distributions for rank and permutation tests |
| ggplot2 | 3.3.0 | Hadley Wickham | Wickham H (2016) Ggplot2: Elegant graphics for data analysis. Springer-Verlag New York |
| kableExtra | 1.1.0 | Hao Zhu | Zhu H (2019) KableExtra: Construct complex table with ’kable’ and pipe syntax |
| knitr | 1.26 | Yihui Xie | Xie Y (2014) Knitr: A comprehensive tool for reproducible research in R. In: Stodden V, Leisch F, Peng RD (eds) Implementing reproducible computational research. Chapman; Hall/CRC  Xie Y (2015) Dynamic documents with R and knitr, 2nd ed. Chapman; Hall/CRC, Boca Raton, Florida  Xie Y (2019) Knitr: A general-purpose package for dynamic report generation in r |
| kSamples | 1.2.9 | Fritz Scholz | Scholz F, Zhu A (2019) KSamples: K-sample rank tests and their combinations |
| lattice | 0.20.38 | Deepayan Sarkar | Sarkar D (2008) Lattice: Multivariate data visualization with r. Springer, New York |
| lemon | 0.4.4 | Stefan McKinnon Edwards | Edwards SM (2020) Lemon: Freshing up your ’ggplot2’ plots |
| pacman | 0.5.1 | Tyler Rinker | Edwards SM (2020) Lemon: Freshing up your ’ggplot2’ plots |
| plyr | 1.8.5 | Hadley Wickham | Wickham H (2011) The split-apply-combine strategy for data analysis. Journal of Statistical Software 40:1–29 |
| Rmisc | 1.5 | Ryan M. Hope | Hope RM (2013) Rmisc: Rmisc: Ryan miscellaneous |
| scales | 1.1.0 | Hadley Wickham | Wickham H, Seidel D (2019) Scales: Scale functions for visualization |
| stringr | 1.4.0 | Hadley Wickham | Wickham H (2019) Stringr: Simple, consistent wrappers for common string operations |
| SuppDists | 1.1.9.5 | ORPHANED | Wheeler B (2020) SuppDists: Supplementary distributions |
| tibble | 2.1.3 | Kirill Muller | Muller K, Wickham H (2019) Tibble: Simple data frames |
| vcd | 1.4.7 | David Meyer | Meyer D, Zeileis A, Hornik K (2006) The strucplot framework: Visualizing multi-way contingency tables with vcd. Journal of Statistical Software 17:1–48  Meyer D, Zeileis A, Hornik K (2020) Vcd: Visualizing categorical data |

Supplementary Table 2; Physiological data, injury intensity and LORR

| **Variable** | **Group** | | | | | **p value** |
| --- | --- | --- | --- | --- | --- | --- |
|  | **SH**  **(n = 4)** | **AS**  **(n = 7)** | **AR**  **(n = 7)** | **CS**  **(n = 7)** | **CR**  **(n = 7)** |  |
| **Age (days)** | 75 ± 3 | 72 ± 2 | 71 ± 2 | 73 ± 3 | 73 ± 2 | 0.784 |
| **Body weight (g)** | 27.7 ± 0.2 | 25.8 ± 0.8 | 27.7 ± 0.4 | 26.8 ± 0.8 | 25.4 ± 0.9 | 0.129 |
| **Physiological parameters during surgery** |  |  |  |  |  |  |
| **Time under anesthesia (min)** | 53.7 ± 7.3 | 57.4 ± 2.9 | 60.0 ± 3.6 | 51.1 ± 3.4 | 58.5 ± 3.2 | 0.394 |
| **Heart rate (bpm)** | 564 ± 5 | 528 ± 15 | 525 ± 13 | 547 ± 10 | 541 ± 14 | 0.178 |
| **Respiratory rate (/min)** | 61 ± 4 | 60 ± 3 | 61 ± 3 | 66 ± 3 | 63 ± 3 | 0.674 |
| **SpO2 (%)** | 95.5 ± 2.1 | 95.2 ± 1.3 | 96.7 ± 0.3 | 96.8 ± 0.4 | 94.8 ± 1.6 | 0.981 |
| **Intensity of Injury (atm)** |  |  |  |  |  |  |
| **1st injury** | (Sham) | 1.58 ± 0.01 | 1.59 ± 0.01 | 1.59 ± 0.01 | 1.57 ± 0.01 |  |
| **2nd injury** | (Sham) | (Sham) | 1.58 ± 0.01 | (Sham) | 1.58 ± 0.01 |  |
| **Duration of LORR (sec)** |  |  |  |  |  |  |
| **1st injury** | 17 ± 2 | 340 ± 48 | 366 ± 28 | 340 ± 26 | 361 ± 38 | 0.012 |
| **2nd injury** | 22 ± 8 | 15 ± 1 | 302 ± 15 | 20 ± 3 | 278 ± 8 | 0.000 |
| Values are presented as “mean ± SEM.”  SH; Sham, AS; acute-phase with single mTBI, AR; acute-phase with repetitive mTBI, CS; chronic-phase with single mTBI, CR; chronic-phase with repetitive mTBI, mTBI; mild traumatic brain injury, LORR; loss of righting reflex, SEM; standard error of the means, SpO2; arterial blood oxygenation | | | | | | |

Supplementary Table 3; Quantification of p-c-Jun+ neurons

|  | **SH** | **AS** | **AR** | **CS** | **CR** |
| --- | --- | --- | --- | --- | --- |
| **Fraction of neuronal population** | 1.50% (1.41-1.60%) | 10.3% (10.1-10.5%) | 10.3% (10.1-10.5%) | 2.85% (2.75-2.95%) | 3.05% (2.95-3.15%) |
| **Statistics and odds ratio** |  |  |  |  |  |
| **vs SH** |  | p < 0.001 (OR = 7.69) | p < 0.001 (OR = 10.0) | p < 0.001 (OR = 1.92) | p < 0.001 (OR = 2.08) |
| **Single vs repetitive mTBI** |  | **AS vs AR** p < 0.001 (OR = 0.77) | | **CS vs CR** p = 0.004 (OR = 0.93) | |
| **Acute vs chronic time point** |  | **AS vs CS** p < 0.001 (OR = 3.92) | | **AR vs CR** p < 0.001 (OR = 4.77) | |
| Values are presented as “mean ± SEM” for neuronal numbers and “Estimate (95% CI)” for fraction of neuronal populations.  SH; Sham, AS; acute-phase with single mTBI, AR; acute-phase with repetitive mTBI, CS; chronic-phase with single mTBI, CR; chronic-phase with repetitive mTBI, mTBI; mild traumatic brain injury, OR; odds ratio, ROI; region of interest, SEM; standard error of the means, CI; confidential interval. | | | | | |

Supplementary Table 4; Mean signal intensity of p-c-Jun

|  | **SH** | **AS** | **AR** | **CS** | **CR** |
| --- | --- | --- | --- | --- | --- |
| **Whole cell body** | 0.140 ± 0.030 | 0.410 ± 0.040 | 0.500 ± 0.030 | 0.180 ± 0.010 | 0.180 ± 0.020 |
| **Statistics and effect size** |  |  |  |  |  |
| **vs SH** |  | p = 0.001 (*r* = 0.88) | p < 0.001 (*r* = 0.93) | p = 0.537 (*r* = 0.40) | p = 0.970 (*r* = 0.21) |
| **Single vs repetitive mTBI** |  | **AS vs AR** p = 0.340 (*r* = 0.45) | | **CS vs CR** p = 0.970 (*r* = 0.03) | |
| **Acute vs chronic time point** |  | **AS vs CS** p = 0.003 (*r* = 0.79) | | **AR vs CR** p = 0.003 (*r* = 0.78) | |
| **Nuclei** | 0.370 ± 0.100 | 1.10 ± 0.080 | 1.30 ± 0.060 | 0.400 ± 0.050 | 0.400 ± 0.070 |
| **Statistics and effect size** |  |  |  |  |  |
| **vs SH** |  | p = 0.008 (*r* = 0.80) | p = 0.004 (*r* = 0.83) | p = 1.00 (*r* = 0.06) | p = 1.00 (*r* = 0.05) |
| **Single vs repetitive mTBI** |  | **AS vs AR** p = 0.149 (*r* = 0.53) | | **CS vs CR** p = 1.00 (*r* = 0.00) | |
| **Acute vs chronic time point** |  | **AS vs CS** p < 0.001 (*r* = 0.89) | | **AR vs CR** p < 0.001 (*r* = 0.93) | |
| **Cytoplasm** | 0.061 ± 0.005 | 0.290 ± 0.020 | 0.370 ± 0.020 | 0.085 ± 0.005 | 0.084 ± 0.007 |
| **Statistics and effect size** |  |  |  |  |  |
| **vs SH** |  | p < 0.001 (*r* = 0.91) | p < 0.001 (*r* = 0.94) | p = 0.050 (*r* = 0.68) | p = 0.075 (*r* = 0.63) |
| **Single vs repetitive mTBI** |  | **AS vs AR** p = 0.077 (*r* = 0.53) | | **CS vs CR** p = 0.920 (*r* = 0.03) | |
| **Acute vs chronic time point** |  | **AS vs CS** p < 0.001 (*r* = 0.84) | | **AR vs CR** p < 0.001 (*r* = 0.90) | |
| Values are presented as “mean ± SEM.”  The values are normalized to the threshold value to determine neuronal positivity for p-c-Jun.  SH; Sham, AS; acute-phase with single mTBI, AR; acute-phase with repetitive mTBI, CS; chronic-phase with single mTBI, CR; chronic-phase with repetitive mTBI, mTBI; mild traumatic brain injury, SEM; standard error of the means. | | | | | |

Supplementary Table 5; Cumulative signal intensity of p-c-Jun

|  | **SH** | **AS** | **AR** | **CS** | **CR** |  |
| --- | --- | --- | --- | --- | --- | --- |
| **Whole cell body** | 283 ± 18 | 1300 ± 87 | 1647 ± 97 | 330 ± 17 | 346 ± 35 |  |
| **Statistics and effect size** |  |  |  |  |  |  |
| **vs SH** |  | p < 0.001 (*r* = 0.93) | p < 0.001 (*r* = 0.94) | p = 0.296 (*r* = 0.49) | p = 0.299 (*r* = 0.43) |  |
| **Single vs repetitive mTBI** |  | **AS vs AR** p = 0.083 (*r* = 0.58) | | **CS vs CR** p = 0.700 (*r* = 0.10) | |  |
| **Acute vs chronic time point** |  | **AS vs CS** p < 0.001 (*r* = 0.87) | | **AR vs CR** p < 0.001 (*r* = 0.91) | |  |
| **Nuclei** | 109 ± 31 | 295 ± 17 | 345 ± 14 | 112 ± 13 | 113 ± 21 |  |
| **Statistics and effect size** |  |  |  |  |  |  |
| **vs SH** |  | p = 0.017 (*r* = 0.77) | p = 0.010 (*r* = 0.80) | p = 1.00 (*r* = 0.02) | p = 1.00 (*r* = 0.03) |  |
| **Single vs repetitive mTBI** |  | **AS vs AR** p = 0.187 (*r* = 0.51) | | **CS vs CR** p = 1.00 (*r* = 0.02) | |  |
| **Acute vs chronic time point** |  | **AS vs CS** p < 0.001 (*r* = 0.91) | | **AR vs CR** p < 0.001 (*r* = 0.91) | |  |
| **Cytoplasm** | 163 ± 15 | 980 ± 69 | 1265 ± 81 | 213 ± 7 | 219 ± 21 |  |
| **Statistics and effect size** |  |  |  |  |  |  |
| **vs SH** |  | p < 0.001 (*r* = 0.93) | p < 0.001 (*r* = 0.94) | p = 0.084 (*r* = 0.59) | p = 0.084 (*r* = 0.64) |  |
| **Single vs repetitive mTBI** |  | **AS vs AR** p = 0.084 (*r* = 0.58) | | **CS vs CR** p = 0.287 (*r* = 0.29) | |  |
| **Acute vs chronic time point** |  | **AS vs CS** p < 0.001 (*r* = 0.87) | | **AR vs CR** p = 0.003 (*r* = 0.78) | |  |
| Values are presented as “mean ± SEM.”  The values are normalized to the threshold value to determine neuronal positivity for p-c-Jun.  SH; Sham, AS; acute-phase with single mTBI, AR; acute-phase with repetitive mTBI, CS; chronic-phase with single mTBI, CR; chronic-phase with repetitive mTBI, mTBI; mild traumatic brain injury, SEM; standard error of the means. | | | | | |  |

Supplementary Table 6; Quantification of NeuroTrace+ cells and NeuN+ cells

|  | **SH** | **AS** | | **AR** | **CS** | **CR** | |
| --- | --- | --- | --- | --- | --- | --- | --- |
| **NeuroTrace+ cells** |  |  | |  |  |  | |
| **Neuronal number (/ROI)** | 1936 ± 105 | 1691 ± 75 | | 1740 ± 100 | 2078 ± 46 | 2120 ± 90 | |
| **Statistics and effect size** |  |  | |  |  |  | |
| **vs SH** |  | p = 1.00 (*r* = 0.48) | | p = 1.00 (*r* = 0.37) | p = 1.00 (*r* = 0.33) | p = 1.00 (*r* = 0.45) | |
| **Single vs repetitive mTBI** |  | **AS vs AR** p = 1.00 (*r* = 0.10) | | | **AS vs AR** p = 1.00 (*r* = 0.27) | | |
| **Acute vs chronic time point** |  | **AS vs CS** p = 0.022 (*r* = 0.74) | | | **AR vs CR** p = 0.197 (*r* = 0.60) | | |
| **NeuN+ cells** |  |  |  | |  | |  |
| **Neuronal number (/ROI)** | 1859 ± 31 | 1497 ± 44 | 1486 ± 44 | | 1812 ± 33 | | 1889 ± 35 |
| **Statistics and effect size** |  |  |  | |  | |  |
| **vs SH** |  | p = 0.097 (*r* = 0.73) | p = 0.001 (*r* = 0.90) | | p = 1.00 (*r* = 0.12) | | p = 1.00 (*r* = 0.19) |
| **Single vs repetitive mTBI** |  | **AS vs AR** p = 1.00 (*r* = 0.11) | | | **AS vs AR** p = 1.00 (*r* = 0.21) | | |
| **Acute vs chronic time point** |  | **AS vs CS** p = 0.011 (*r* = 0.78) | | | **AR vs CR** p < 0.001 (*r* = 0.88) | | |
| **NeuroTrace+ vs NeuN+ in number** | p = 1.00 (*r* = 0.21) | p = 0.14 (*r* = 0.63) | p = 0.13 (*r* = 0.64) | | p = 0.02 (*r* = 0.75) | | p = 0.38 (*r* = 0.55) |
| Values are presented as “mean ± SEM” for neuronal numbers and “Estimate (95% CI)” for fraction of neuronal populations.  SH; Sham, AS; acute-phase with single mTBI, AR; acute-phase with repetitive mTBI, CS; chronic-phase with single mTBI, CR; chronic-phase with repetitive mTBI, mTBI; mild traumatic brain injury, ROI; region of interest, SEM; standard error of the means. | | | | | | | |

Supplementary Table 7; Fraction of NeuN+ neurons

|  | **SH** | **AS** | **AR** | **CS** | **CR** |
| --- | --- | --- | --- | --- | --- |
| **Fraction of NeuN+ neurons in**  **NeuroTrace+ neurons** | 87.3% (87.1-87.6%) | 81.7% (81.5-82.0%) | 79.9% (79.7-80.2%) | 79.8% (79.5-80.0%) | 81.7% (81.5-82.0%) |
| **Statistics and odds ratio** |  |  |  |  |  |
| **vs SH** |  | p < 0.001 (OR = 0.65) | p < 0.001 (OR = 0.58) | p < 0.001 (OR = 0.57) | p < 0.001 (OR = 0.65) |
| **Single vs repetitive mTBI** |  | **AS vs AR** p < 0.001 (OR = 1.13) | | **CS vs CR** p < 0.001 (OR = 0.879) | |
| **Acute vs chronic time point** |  | **AS vs CS** p < 0.001 (OR = 1.14) | | **AR vs CR** p < 0.001 (OR = 0.888) | |
| **Fraction in p-c-Jun+ neurons** | 88.9% (86.8-90.9%) | 89.1% (88.4-89.7%) | 90.7% (90.2-91.2%) | 78.0% (76.5-79.4%) | 78.4% (77.0-79.7%) |
| **Fraction in p-c-Jun- neurons** | 87.3% (87.1-87.6%) | 80.9% (80.6-81.2%) | 78.3% (78.0-78.6%) | 79.8% (79.6-80.0%) | 81.9% (81.6-82.1%) |
| **Statistics and effect size** |  |  |  |  |  |
| **p-c-Jun+ vs p-c-Jun- neurons** | p = 0.415 (OR = 1.17) | p < 0.001 (OR = 1.92) | p <0.001 (OR = 2.70) | p = 0.048 (OR = 0.90) | p < 0.001 (OR = 0.80) |
| **Within p-c-Jun+ neurons** |  |  |  |  |  |
| **vs SH** |  | p = 1.00 (OR = 1.01) | p = 0.315 (OR = 1.21) | p <0.001 (OR = 0.441) | p <0.001 (OR = 0.450) |
| **Single vs repetitive mTBI** |  | **AS vs AR** p <0.001 (OR = 0.835) | | **CS vs CR** p = 1.00 (OR = 0.978) | |
| **Acute vs chronic time point** |  | **AS vs CS** p <0.001 (OR = 2.297) | | **AR vs CR** p <0.001 (OR = 2.692) | |
| **Within p-c-Jun- neurons** |  |  |  |  |  |
| **vs SH** |  | p < 0.001 (OR = 0.613) | p < 0.001 (OR = 0.524) | p < 0.001 (OR = 0.574) | p < 0.001 (OR = 0.655) |
| **Single vs repetitive mTBI** |  | **AS vs AR** p <0.001 (OR = 1.175) | | **CS vs CR** p <0.001 (OR = 0.877) | |
| **Acute vs chronic time point** |  | **AS vs CS** p <0.001 (OR = 1.072) | | **AR vs CR** p <0.001 (OR = 0.800) | |
| Values are presented as “Estimate (95% CI).” SH; Sham, AS; acute-phase with single mTBI, AR; acute-phase with repetitive mTBI, CS; chronic-phase with single mTBI, CR; chronic-phase with repetitive mTBI, mTBI; mild traumatic brain injury, OR; odds ratio, ROI; region of interest, SEM; standard error of the means, CI; confidential interval. | | | | | |

Supplementary Table 8; Mean signal intensity of NeuN

|  | **SH** | **AS** | **AR** | **CS** | **CR** |
| --- | --- | --- | --- | --- | --- |
| **Whole neuronal population** | 0.988 ± 0.0376 | 0.958 ± 0.0403 | 1.05 ± 0.0357 | 0.932 ± 0.0266 | 0.912 ± 0.0219 |
| **Statistics and effect size** |  |  |  |  |  |
| **vs SH** |  | p = 1.00 (*r* = 0.16) | p = 1.00 (*r* = 0.33) | p = 1.00 (*r* = 0.33) | p = 0.860 (*r* = 0.44) |
| **Single vs repetitive mTBI** |  | **AS vs AR** p = 0.837 (*r* = 0.41) | | **CS vs CR** p = 1.00 (*r* = 0.15) | |
| **Acute vs chronic time point** |  | **AS vs CS** p = 1.00 (*r* = 0.14) | | **AR vs CR** p = 0.071 (*r* = 0.64) | |
| **p-c-Jun+ neurons** | 1.00 ± 0.047 | 0.968 ± 0.018 | 0.993 ± 0.028 | 1.00 ± 0.049 | 0.987 ± 0.035 |
| **p-c-Jun- neurons** | 1.01 ± 0.015 | 0.949 ± 0.017 | 0.988 ± 0.026 | 0.991 ± 0.028 | 0.990 ± 0.018 |
| **Statistics and effect size** |  |  |  |  |  |
| **p-c-Jun+ vs p-c-Jun- neurons** | p = 1.00 (*r* = 0.07) | p = 1.00 (*r* = 0.37) | p = 1.00 (*r* = 0.06) | p = 1.00 (*r* = 0.12) | p = 1.00 (*r* = 0.05) |
| **Within p-c-Jun+ neurons** |  |  |  |  |  |
| **vs SH** |  | p = 1.00 (*r* = 0.19) | p = 1.00 (*r* = 0.05) | p = 1.00 (*r* = 0.02) | p = 1.00 (*r* = 0.07) |
| **Single vs repetitive mTBI** |  | **AS vs AR** p = 1.00 (*r* = 0.20) | | **CS vs CR** p = 1.00 (*r* = 0.08) | |
| **Acute vs chronic time point** |  | **AS vs CS** p = 1.00 (*r* = 0.18) | | **AR vs CR** p = 1.00 (*r* = 0.04) | |
| **Within p-c-Jun- neurons** |  |  |  |  |  |
| **vs SH** |  | p = 0.557 (*r* = 0.62) | p = 1.00 (*r* = 0.22) | p = 1.00 (*r* = 0.18) | p = 1.00 (*r* = 0.26) |
| **Single vs repetitive mTBI** |  | **AS vs AR** p = 1.00 (*r* = 0.31) | | **CS vs CR** p = 1.00 (*r* = 0.01) | |
| **Acute vs chronic time point** |  | **AS vs CS** p = 1.00 (*r* = 0.32) | | **AR vs CR** p = 1.00 (*r* = 0.02) | |
| Values are presented as “mean ± SEM.”  “Whole neuronal population”; values are averaged for single neurons and normalized to the median of SH group  “p-c-Jun+ neurons” and “p-c-Jun- neurons”; values are averaged for single neurons and normalized to the median of p-c-Jun- neurons in SH group.  SH; Sham, AS; acute-phase with single mTBI, AR; acute-phase with repetitive mTBI, CS; chronic-phase with single mTBI, CR; chronic-phase with repetitive mTBI, mTBI; mild traumatic brain injury, SEM; standard error of the means. | | | | | |

Supplementary Table 9; Cumulative signal intensity of NeuN

|  | **SH** | **AS** | **AR** | **CS** | **CR** |
| --- | --- | --- | --- | --- | --- |
| **p-c-Jun+ neurons** | 1.12 ± 0.103 | 1.25 ± 0.095 | 1.29 ± 0.059 | 1.07 ± 0.059 | 1.02 ± 0.069 |
| **p-c-Jun- neurons** | 0.989 ± 0.041 | 0.949 ± 0.040 | 1.04 ± 0.041 | 0.942 ± 0.029 | 0.917 ± 0.023 |
| **Statistics and effect size** |  |  |  |  |  |
| **p-c-Jun+ vs p-c-Jun- neurons** | p = 1.00 (*r* = 0.42) | p = 0.297 (*r* = 0.60) | p = 0.033 (*r* = 0.60) | p = 0.701 (*r* = 0.52) | p = 1.00 (*r* = 0.34) |
| **Within p-c-Jun+ neurons** |  |  |  |  |  |
| **vs SH** |  | p = 1.00 (*r* = 0.27) | p = 1.00 (*r* = 0.38) | p = 1.00 (*r* = 0.11) | p = 1.00 (*r* = 0.23) |
| **Single vs repetitive mTBI** |  | **AS vs AR** p = 1.00 (*r* = 0.11) | | **CS vs CR** p = 1.00 (*r* = 0.15) | |
| **Acute vs chronic time point** |  | **AS vs CS** p = 1.00 (*r* = 0.38) | | **AR vs CR** p = 0.227 (*r* = 0.62) | |
| **Within p-c-Jun- neurons** |  |  |  |  |  |
| **vs SH** |  | p = 1.00 (*r* = 0.21) | p = 1.00 (*r* = 0.26) | p = 1.00 (*r* = 0.26) | p = 1.00 (*r* = 0.39) |
| **Single vs repetitive mTBI** |  | **AS vs AR** p = 1.00 (*r* = 0.39) | | **CS vs CR** p = 1.00 (*r* = 0.18) | |
| **Acute vs chronic time point** |  | **AS vs CS** p = 1.00 (*r* = 0.04) | | **AR vs CR** p = 0.483 (*r* = 0.56) | |
| Values are presented as “mean ± SEM.”  Values are averaged for single neurons and normalized to the median of p-c-Jun- neurons in SH group.  SH; Sham, AS; acute-phase with single mTBI, AR; acute-phase with repetitive mTBI, CS; chronic-phase with single mTBI, CR; chronic-phase with repetitive mTBI, mTBI; mild traumatic brain injury, SEM; standard error of the means. | | | | | |

Supplementary Table 10; Cellular volume of neurons by injury mode

|  | **SH** | **AS** | **AR** | **CS** | **CR** |
| --- | --- | --- | --- | --- | --- |
| **Whole neuronal population (μm^3^)** | 474 ± 17 | 487 ± 17 | 512 ± 10 | 452 ± 11 | 447 ± 7 |
| **Statistics and effect size** |  |  |  |  |  |
| **vs SH** |  | p = 1.00 (*r* = 0.05) | p = 0.255 (*r* = 0.60) | p = 1.00 (*r* = 0.26) | p = 1.00 (*r* = 0.37) |
| **Single vs repetitive mTBI** |  | **AS vs AR** p = 1.00 (*r* = 0.32) | | **CS vs CR** p = 1.00 (*r* = 0.10) | |
| **Acute vs chronic time point** |  | **AS vs CS** p = 0.670 (*r* = 0.42) | | **AR vs CR** p = 0.002 (*r* = 0.81) | |
| **p-c-Jun+ neurons (μm^3^)** | 545 ± 51 | 626 ± 36 | 634 ± 17 | 487 ± 24 | 506 ± 28 |
| **p-c-Jun- neurons (μm^3^)** | 473 ± 16 | 474 ± 16 | 493 ± 11 | 451 ± 11 | 445 ± 7 |
| **Statistics and effect size** |  |  |  |  |  |
| **p-c-Jun+ vs p-c-Jun- neurons** | p = 1.00 (*r* = 0.46) | p = 0.02 (*r* = 0.76) | p <0.001 (*r* = 0.88) | p = 1.00 (*r* = 0.47) | p = 1.00 (*r* = 0.46) |
| **Within p-c-Jun+ neurons** |  |  |  |  |  |
| **vs SH** |  | p = 1.00 (*r* = 0.36) | p = 1.00 (*r* = 0.40) | p = 1.00 (*r* = 0.28) | p = 1.00 (*r* = 0.19) |
| **Single vs repetitive mTBI** |  | **AS vs AR** p = 1.00 (*r* = 0.05) | | **CS vs CR** p = 1.00 (*r* = 0.13) | |
| **Acute vs chronic time point** |  | **AS vs CS** p = 0.145 (*r* = 0.64) | | **AR vs CR** p = 0.057 (*r* = 0.70) | |
| **Within p-c-Jun- neurons** |  |  |  |  |  |
| **vs SH** |  | p = 1.00 (*r* = 0.12) | p = 1.00 (*r* = 0.11) | p = 1.00 (*r* = 0.31) | p = 1.00 (*r* = 0.37) |
| **Single vs repetitive mTBI** |  | **AS vs AR** p = 1.00 (*r* = 0.27) | | **CS vs CR** p = 1.00 (*r* = 0.13) | |
| **Acute vs chronic time point** |  | **AS vs CS** p = 1.00 (*r* = 0.30) | | **AR vs CR** p = 0.057 (*r* = 0.70) | |
| **NeuN+ neurons (μm^3^)** | 496 ± 18 | 520 ± 18 | 550 ± 10 | 482 ± 13 | 473 ± 8 |
| **NeuN- neurons (μm^3^)** | 317 ± 17 | 338 ± 21 | 353 ± 14 | 335 ± 10 | 328 ± 12 |
| **Statistics and effect size** |  |  |  |  |  |
| **NeuN+ vs NeuN- neurons** | p = 0.003 (*r* = 0.95) | p <0.001 (*r* = 0.92) | p <0.001 (*r* = 0.93) | p <0.001 (*r* = 0.90) | p <0.001 (*r* = 0.93) |
| **Within NeuN+ neurons** |  |  |  |  |  |
| **vs SH** |  | p = 1.00 (*r* = 0.27) | p = 0.754 (*r* = 0.56) | p = 1.00 (*r* = 0.18) | p = 1.00 (*r* = 0.31) |
| **Single vs repetitive mTBI** |  | **AS vs AR** p = 1.00 (*r* = 0.36) | | **CS vs CR** p = 1.00 (*r* = 0.15) | |
| **Acute vs chronic time point** |  | **AS vs CS** p = 1.00 (*r* = 0.42) | | **AR vs CR** p = 0.001 (*r* = 0.85) | |
| **Within NeuN- neurons** |  |  |  |  |  |
| **vs SH** |  | p = 1.00 (*r* = 0.24) | p = 1.00 (*r* = 0.32) | p = 1.00 (*r* = 0.26) | p = 1.00 (*r* = 0.16) |
| **Single vs repetitive mTBI** |  | **AS vs AR** p = 1.00 (*r* = 0.28) | | **CS vs CR** p = 1.00 (*r* = 0.12) | |
| **Acute vs chronic time point** |  | **AS vs CS** p = 1.00 (*r* = 0.04) | | **AR vs CR** p = 1.00 (*r* = 0.29) | |
| Values are presented as “mean ± SEM ($\mu m^{3}$).” SH; Sham, AS; acute-phase with single mTBI, AR; acute-phase with repetitive mTBI, CS; chronic-phase with single mTBI, CR; chronic-phase with repetitive mTBI, mTBI; mild traumatic brain injury, SEM; standard error of the means. | | | | | |

Supplementary Table 11; Nuclear volume of neurons by injury mode

|  | **SH** | **AS** | **AR** | **CS** | **CR** |
| --- | --- | --- | --- | --- | --- |
| **Whole neuronal population (μm^3^)** | 56 ± 0.7 | 56 ± 0.6 | 55 ± 0.4 | 56 ± 0.5 | 56 ± 0.4 |
| **Statistics and effect size** |  |  |  |  |  |
| **vs SH** |  | p = 1.00 (*r* = 0.10) | p = 0.937 (*r* = 0.45) | p = 1.00 (*r* = 0.11) | p = 1.00 (*r* = 0.20) |
| **Single vs repetitive mTBI** |  | **AS vs AR** p = 1.00 (*r* = 0.08) | | **CS vs CR** p = 1.00 (*r* = 0.05) | |
| **Acute vs chronic time point** |  | **AS vs CS** p = 1.00 (*r* = 0.17) | | **AR vs CR** p = 0.937 (*r* = 0.41) | |
| **p-c-Jun+ neurons (μm^3^)** | 61 ± 0.8 | 59 ± 0.6 | 58 ± 0.5 | 62 ± 1 | 61 ± 1 |
| **p-c-Jun- neurons (μm^3^)** | 56 ± 0.7 | 55 ± 0.6 | 54 ± 0.4 | 56 ± 0.5 | 56 ± 0.5 |
| **Statistics and effect size** |  |  |  |  |  |
| **p-c-Jun+ vs p-c-Jun- neurons** | p = 0.057 (*r* = 0.56) | p = 0.006 (*r* = 0.80) | p <0.001 (*r* = 0.86) | p = 0.007 (*r* = 0.79) | p = 0.007 (*r* = 0.79) |
| **Within p-c-Jun+ neurons** |  |  |  |  |  |
| **vs SH** |  | p = 0.765 (*r* = 0.54) | p = 0.170 (*r* = 0.70) | p = 1.00 (*r* = 0.14) | p = 1.00 (*r* = 0) |
| **Single vs repetitive mTBI** |  | **AS vs AR** p = 1.00 (*r* = 0.39) | | **CS vs CR** p = 1.00 (*r* = 0.12) | |
| **Acute vs chronic time point** |  | **AS vs CS** p = 0.762 (*r* = 0.49) | | **AR vs CR** p = 0.170 (*r* = 0.62) | |
| **Within p-c-Jun- neurons** |  |  |  |  |  |
| **vs SH** |  | p = 1.00 (*r* = 0.35) | p = 0.810 (*r* = 0.35) | p = 1.00 (*r* = 0.14) | p = 1.00 (*r* = 0.16) |
| **Single vs repetitive mTBI** |  | **AS vs AR** p = 1.00 (*r* = 0.21) | | **CS vs CR** p = 1.00 (*r* = 0.02) | |
| **Acute vs chronic time point** |  | **AS vs CS** p = 1.00 (*r* = 0.27) | | **AR vs CR** p = 0.503 (*r* = 0.53) | |
| **NeuN+ neurons (μm^3^)** | 57 ± 0.7 | 56 ± 0.6 | 55 ± 0.4 | 56 ± 0.5 | 56 ± 0.4 |
| **NeuN- neurons (μm^3^)** | 52 ± 1 | 54 ± 1 | 52 ± 0.4 | 54 ± 0.7 | 53 ± 0.4 |
| **Statistics and effect size** |  |  |  |  |  |
| **NeuN+ vs NeuN- neurons** | p = 0.096 (*r* = 0.84) | p = 0.531 (*r* = 0.55) | p = 0.008 (*r* = 0.79) | p = 0.281 (*r* = 0.60) | p <0.001 (*r* = 0.88) |
| **Within NeuN+ neurons** |  |  |  |  |  |
| **vs SH** |  | p = 1.00 (*r* = 0.10) | p = 1.00 (*r* = 0.42) | p = 1.00 (*r* = 0.15) | p = 1.00 (*r* = 0.20) |
| **Single vs repetitive mTBI** |  | **AS vs AR** p = 1.00 (*r* = 0.08) | | **CS vs CR** p = 1.00 (*r* = 0.04) | |
| **Acute vs chronic time point** |  | **AS vs CS** p = 1.00 (*r* = 0.17) | | **AR vs CR** p = 1.00 (*r* = 0.45) | |
| **Within NeuN- neurons** |  |  |  |  |  |
| **vs SH** |  | p = 1.00 (*r* = 0.20) | p = 1.00 (*r* = 0.03) | p = 1.00 (*r* = 0.42) | p = 1.00 (*r* = 0.23) |
| **Single vs repetitive mTBI** |  | **AS vs AR** p = 1.00 (*r* = 0.23) | | **CS vs CR** p = 1.00 (*r* = 0.33) | |
| **Acute vs chronic time point** |  | **AS vs CS** p = 1.00 (*r* = 0.21) | | **AR vs CR** p = 1.00 (*r* = 0.34) | |
| Values are presented as “mean ± SEM ($\mu m^{3}$).” SH; Sham, AS; acute-phase with single mTBI, AR; acute-phase with repetitive mTBI, CS; chronic-phase with single mTBI, CR; chronic-phase with repetitive mTBI, Ex-S; extra-small, S; small, M; middle, L; large, Ex-L; extra-large, mTBI; mild traumatic brain injury, SEM; standard error of the means. | | | | | |

Supplementary Table 12; Fraction of p-c-Jun+ neurons depending on cellular volume

|  | **SH** | **AS** | **AR** | **CS** | **CR** |
| --- | --- | --- | --- | --- | --- |
| **p-c-Jun+ neuronal fraction** |  |  |  |  |  |
| **Ex-S** | 1.19% (1.00-1.39%) | 6.43% (6.08-6.79%) | 7.93% (7.55-8.32%) | 2.41% (2.21-2.61%) | 2.07% (1.89-2.26%) |
| **S** | 1.35% (1.15-1.57%) | 8.22% (7.83-8.62%) | 10.4% (10.0-10.9%) | 2.63% (2.42-2.84%) | 2.61% (2.41-2.82%) |
| **M** | 1.44% (1.24-1.67%) | 9.99% (9.56-10.4%) | 12.7% (12.2-13.2%) | 2.77% (2.56-2.99%) | 2.94% (2.73-3.16%) |
| **L** | 1.56% (1.35-1.79%) | 11.4% (10.9-11.8%) | 14.5% (14.0-15.0%) | 2.97% (2.76-3.20%) | 3.45% (3.23-3.69%) |
| **Ex-L** | 1.98% (1.75-2.25%) | 15.5% (15.0-16.0%) | 19.6% (19.0-20.2%) | 3.47% (3.24-3.71%) | 4.18% (3.92-4.44%) |
| **Inter-subgroup comparison** |  |  |  |  |  |
| **Ex-S vs S** | p = 0.789 (OR = 1.14) | p < 0.001 (OR = 1.28) | p < 0.001 (OR = 1.32) | p = 0.436 (OR = 1.09) | p < 0.001 (OR = 1.26) |
| **vs M** | p = 0.400 (OR = 1.22) | p < 0.001 (OR = 1.55) | p < 0.001 (OR = 1.60) | p = 0.087 (OR = 1.15) | p < 0.001 (OR = 1.42) |
| **vs L** | p = 0.086 (OR = 1.31) | p < 0.001 (OR = 1.77) | p < 0.001 (OR = 1.83) | p = 0.002 (OR = 1.23) | p < 0.001 (OR = 1.67) |
| **vs Ex-L** | p < 0.001 (OR = 2.67) | p < 0.001 (OR = 2.41) | p < 0.001 (OR = 2.47) | p < 0.001 (OR = 1.44) | p < 0.001 (OR = 2.02) |
| **S vs M** | p = 0.937 (OR = 1.07) | p < 0.001 (OR = 1  .22) | p < 0.001 (OR = 1.22) | p = 0.436 (OR = 1.05) | p = 0.032 (OR = 1.13) |
| **vs L** | p = 0.700 (OR = 1.16) | p < 0.001 (OR = 1.38) | p < 0.001 (OR = 1.39) | p = 0.106 (OR = 1.13) | p < 0.001 (OR = 1.32) |
| **vs Ex-L** | p = 0.001 (OR = 1.47) | p < 0.001 (OR = 1.89) | p < 0.001 (OR = 1.88) | p < 0.001 (OR = 1.32) | p < 0.001 (OR = 1.60) |
| **M vs L** | p = 0.937 (OR = 1.08) | p < 0.001 (OR = 1.14) | p < 0.001 (OR = 1.14) | p = 0.436 (OR = 1.07) | p = 0.004 (OR = 1.17) |
| **vs Ex-L** | p = 0.010 (OR = 1.37) | p < 0.001 (OR = 1.55) | p < 0.001 (OR = 1.54) | p < 0.001 (OR = 1.25) | p < 0.001 (OR = 1.42) |
| **L vs Ex-L** | p = 0.086 (OR = 1.27) | p < 0.001 (OR = 1.36) | p < 0.001 (OR = 1.35) | p = 0.020 (OR = 1.17) | p < 0.001 (OR = 1.21) |
| Values are presented as “Estimate (95% CI).” SH; Sham, AS; acute-phase with single mTBI, AR; acute-phase with repetitive mTBI, CS; chronic-phase with single mTBI, CR; chronic-phase with repetitive mTBI, mTBI; mild traumatic brain injury, Ex-S; extra-small, S; small, M; middle, L; large,  Ex-L; extra-large, OR; odds ratio, ROI; region of interest, SEM; standard error of the means, CI; confidential interval. | | | | | |

Supplementary Table 13; Fraction of NeuN+ neurons depending on cellular volume

|  | **SH** | **AS** | **AR** | **CS** | **CR** |
| --- | --- | --- | --- | --- | --- |
| **NeuN+ neuronal fraction** |  |  |  |  |  |
| **Ex-S** | 74.9% (74.1-75.6%) | 66.0% (65.3-66.7%) | 63.3% (62.6-64.0%) | 65.5% (64.9-66.1%) | 68.1% (67.5-68.6%) |
| **S** | 85.6% (85.0-86.2%) | 78.2% (77.6-78.8%) | 76.3% (75.7-77.0%) | 76.5% (76.0-77.1%) | 79.0% (78.5-79.5%) |
| **M** | 89.7% (89.2-90.3%) | 84.4% (83.9-84.9%) | 82.7% (82.2-83.3%) | 81.9% (81.4-82.4%) | 83.9% (83.5-84.4%) |
| **L** | 92.0% (91.5-92.4%) | 88.4% (88.0-88.9%) | 86.8% (86.3-87.3%) | 85.5% (85.1-86.0%) | 87.3% (86.9-87.8%) |
| **Ex-L** | 94.6% (94.1-95.0%) | 91.7% (91.3-92.0%) | 90.4% (90.0-90.8%) | 89.3% (88.9-89.7%) | 90.4% (90.0-90.8%) |
| **Inter-subgroup comparison** |  |  |  |  |  |
| **Ex-S vs S** | p < 0.001 (OR = 1.14) | p < 0.001 (OR = 1.18) | p < 0.001 (OR = 1.21) | p < 0.001 (OR = 1.17) | p < 0.001 (OR = 1.16) |
| **vs M** | p < 0.001 (OR = 1.20) | p < 0.001 (OR = 1.28) | p < 0.001 (OR = 1.31) | p < 0.001 (OR = 1.25) | p < 0.001 (OR = 1.23) |
| **vs L** | p < 0.001 (OR = 1.23) | p < 0.001 (OR = 1.34) | p < 0.001 (OR = 1.37) | p < 0.001 (OR = 1.31) | p < 0.001 (OR = 1.28) |
| **vs Ex-L** | p < 0.001 (OR = 1.26) | p < 0.001 (OR = 1.39) | p < 0.001 (OR = 1.43) | p < 0.001 (OR = 1.36) | p < 0.001 (OR = 1.33) |
| **S vs M** | p = 0.036 (OR = 1.05) | p < 0.001 (OR = 1.08) | p < 0.001 (OR = 1.08) | p < 0.001 (OR = 1.07) | p < 0.001 (OR = 1.06) |
| **vs L** | p < 0.001 (OR = 1.07) | p < 0.001 (OR = 1.13) | p < 0.001 (OR = 1.14) | p < 0.001 (OR = 1.12) | p < 0.001 (OR = 1.11) |
| **vs Ex-L** | p < 0.001 (OR = 1.10) | p < 0.001 (OR = 1.17) | p < 0.001 (OR = 1.18) | p < 0.001 (OR = 1.17) | p < 0.001 (OR = 1.11) |
| **M vs L** | p = 0.255 (OR = 1.02) | p = 0.004 (OR = 1.05) | p = 0.003 (OR = 1.05) | p = 0.003 (OR = 1.04) | p = 0.007 (OR = 1.04) |
| **vs Ex-L** | p = 0.017 (OR = 1.05) | p < 0.001 (OR = 1.09) | p < 0.001 (OR = 1.09) | p < 0.001 (OR = 1.09) | p < 0.001 (OR = 1.08) |
| **L vs Ex-L** | p = 0.255 (OR = 1.03) | p = 0.017 (OR = 1.04) | p = 0.007 (OR = 1.04) | p = 0.003 (OR = 1.04) | p = 0.010 (OR = 1.03) |
| Values are presented as “Estimate (95% CI).” SH; Sham, AS; acute-phase with single mTBI, AR; acute-phase with repetitive mTBI, CS; chronic-phase with single mTBI, CR; chronic-phase with repetitive mTBI, mTBI; mild traumatic brain injury, Ex-S; extra-small, S; small, M; middle, L; large,  Ex-L; extra-large, OR; odds ratio, ROI; region of interest, SEM; standard error of the means, CI; confidential interval. | | | | | |
